# Supplementary material for: Thermoresponsive polymer assemblies via variable temperature liquid-phase transmission electron microscopy and small angle X-ray scattering
Source: Nat Commun. 2021 Nov 12;12:6568. doi: 10.1038/s41467-021-26773-z (PMC8589985; doi:10.1038/s41467-021-26773-z)
Supplement: Supplementary file 1 — Supplementary Information [file 41467_2021_26773_MOESM1_ESM.pdf]

Supporting Information for

Thermoresponsive Polymer Assemblies via Variable Temperature Liquid-Phase Transmission Electron Microscopy and Small Angle X-Ray Scattering

Joanna Korpanty,[1] Lucas R. Parent,[2] Nicholas Hampu,[1] Steven Weigand,[3] and Nathan C. Gianneschi[1,4,5,6]*

[1]Department of Chemistry, International Institute for Nanotechnology, Chemistry of Life Processes Institute, Simpson Querrey Institute, Northwestern University, Evanston, Illinois 60208, United States

[2]Innovation Partnership Building, University of Connecticut, Storrs, CT 06269, USA.

[3]DuPont−Northwestern−Dow Collaborative Access Team (DND-CAT) Synchrotron Research Center, Northwestern University, Argonne, Illinois 60208, United States

[4]Department of Materials Science & Engineering, Northwestern University, Evanston, Illinois 60208, United States

[5]Department of Biomedical Engineering, Northwestern University, Evanston, Illinois 60208, United States

[6]Department of Biomedical Engineering and Department of Pharmacology, Northwestern University, Evanston, Illinois 60208, United States

*Corresponding author: nathan.gianneschi@northwestern.edu

Contents

I. Additional LCTEM Imaging Details

II. Imaging Processing Details

III. DLS

IV. SEC-MALS

V. ^{1}H NMR

VI. Additional VT-SAXS Details

VII. Supporting Figures

VIII. Synthesis

IX. Kinetics Experiments

X. Supplementary References

I. Additional LCTEM Imaging Details

Two VT-LCTEM experiments (Supplementary Figures 19-20) were performed using a Thermo Fisher Titan Themis TEM (Thermo Fisher Scientific, Waltham, MA) at the UConn Center for Advanced Microscopy and Materials Analysis (CAMMA) operated at 300 keV (gun lens: 4, spot size: 10, CLA(2): 150 μm, beam current: 0.296 nA measured through vacuum, and beam diameter: 14.7 μm). Micrographs were recorded on a 4k × 4k Thermo Fisher Ceta-16M CMOS camera (binning: 2) using Thermo Fisher TIA image acquisition software (Thermo Fisher Scientific, Waltham, MA).

One VT-LCTEM experiment (Supplementary Figure 21) was performed using a Thermo Fisher Talos TEM (Thermo Fisher Scientific, Waltham, MA) at the UConn Center for Advanced Microscopy and Materials Analysis (CAMMA) operated at 200 keV (gun lens: 3, spot size: 7, CLA(2): 100 μm, beam current: 0.277 nA measured through vacuum, and beam diameter: 14.7 μm). Micrographs were recorded on a 4k × 4k Thermo Fisher Ceta-16M CMOS camera (binning: 2) using Thermo Fisher TIA image acquisition software (Thermo Fisher Scientific, Waltham, MA).

II. Image Processing Details

To probe the morphological transformation of the triblock further, we performed image processing using the software FIJI. First, we cropped a region of interest in a fixed area for each

timepoint (Figure 4d). We binned the cropped images (2x2 average), applied a gaussian filter (σ = 1), thresholded each cropped region of interest, and subtracted features less than 5 pixels^2 from each image (Figure 4e). The intermediate contrast of the corona was better captured using a Lookup Table (LUT), whereby each pixel value range was assigned a corresponding red, green, and blue value. Using a 6-shade LUT enabled colorization and thus allowed for a clearer visual distinction between multiple features with different intensity values (Figure 4f).

For the MATLAB image processing showed in Supplementary Figures 9 and 14, we cropped each image to only feature the electron transparent SiN_x membrane, and we enhanced the images with imadjust, histeq, and adapthisteq, which are functions available with the image processing toolkit in MATLAB.

III. DLS

Dynamic light scattering (DLS) measurements were performed on a Zetasizer (Malvern Instruments Ltd, Nano ZS) with a 120 second equilibrium time for each measurement.

IV. SEC-MALS

Polymers were dissolved at 5 mg mL^{-1} in DMF and analyzed by size exclusion chromatography multiangle light scattering (SEC-MALS) with on a Phenomenex Phenogel 5u 103Å, 1K-75K, 300 x 7.8 mm in series with a Phenomenex Phenogel 5u 103Å, 10K-100K, 300 x 7.80 mm) at 65 ° C in 0.05 M LiBr in DMF, using a ChromTech Series 1500 pump equipped with a multi-angle light scattering detector (DAWNHELIOS II, Wyatt Technology) and a refractive index detector (Wyatt Optilab T-rEX) normalized to a 30000 MW polystyrene standard at a flow rate of 0.75 mL/min.

V. ^{1}H NMR

Proton Nuclear Magnetic Resonance Spectroscopy (^{1}H NMR) experiments were all performed on a Bruker Advance III HD system equipped with a TXO Prodigy probe.

VI. Additional VT-SAXS Details

The scattering length densities of PHPMA and water are quite similar, especially since the PHPMA core is likely moderately hydrated,[1] which may cause relatively low scattering contrast for the SAXS trace at 30 °C before heating. Additionally, weak, yet still existent, spatial

correlations between the PDEGMA and PHPMA block may smear the overall scattering pattern and obscure characteristic features commonly observed for micelles. Finally, the small diameter of these micelles, c.a. 12 nm radius by DLS, suggests that the features characteristic of a spherical form factor would be pushed to the edge of the SAXS detector.[2]

Data was fit to well-defined form factor models using the SASView software (http://www.sasview.org/). With the assumption that the triblock assembles into a spherical micelle with a PHPMA core, a PDEGMA inner shell, and a PEG outer shell below the LCST of PDEGMA, data obtained at 30 °C was fit to a core-shell-shell sphere form factor:

$$P(q) = \frac{3V_{core}}{qR_{core}}(\rho_{core} - \rho_{inner})J_{core}(qR_{core}) + \frac{3V_{inner}}{qR_{inner}}(\rho_{inner} - \rho_{outer})J_{inner}(qR_{inner}) + \frac{3V_{outer}}{qR_{outer}}(\rho_{solvent} - \rho_{outer})J_{outer}(qR_{outer})$$

where R is the radius of the core or thickness of the shells, V is the volume of the core or shell ($V = 4\pi R^3/3$), ρ is the scattering length density, and J is a first order Bessel function of the form

$$J = \frac{\sin(x) - x\cos(x)}{x^2}$$

During the fitting process, the inner and outer shell thicknesses were fixed after estimating based on known degrees of polymerization, statistical segment lengths, and assuming scaling for a good solvent. Solvent SLD, the incoherent background intensity, and the log-normal size distributions of the core, inner shell, and outer shell were also fixed. All other parameters were fit using SASView. Extracted fit parameters as well as fixed parameters are included below (Supplementary Table 1). Bolded values indicate that these parameters were extracted during the fitting process. The fit deviates from the experimental data at low q, suggesting the presence of loosely formed aggregates.

Supplementary Table 1. Extracted Fitting Parameters for at 30 °C SAXS Trace

Parameter	Value
Intensity Pre-factor	**0.03**
Background	0.01
SLD_{PHPMA}	**22.9E-6 A^{-2}**
Core Radius	**18 nm**
SLD_{PDEGMA}	**25.9E-6 A^{-2}**
Inner Shell Thickness	1.5 nm
SLD_{PEG}	**25.8E-6 A^{-2}**
Outer Shell Thickness	4.2 nm

SLD_{H2O}	26.1E-6 A^{-2}
Core Size Distribution	0.3
Shell Size Distribution	0.3
Corona Size Distribution	0.3

We attempted to fit the scattering data acquired above the LCST at 60 °C to the same core-shell-shell sphere form factor with the assumption that the hydrated PDEGMA block at 30 °C becomes hydrophobic and collapses into the micelle core upon crossing its LCST. The short length of the PDEGMA block as compared to the PHPMA block paired with the persistence of the scattering features observed at 60 °C upon cooling back to 30 °C suggests that PDEGMA resides within the interior of the micelle core and is surrounded by a PHPMA shell. The PEG outer shell remains hydrophilic and extended into the aqueous solvent. Using these assumptions, we attempted to fit the 60 °C scattering data to the core-shell-shell sphere model where PDEGMA is the micelle core, PHPMA is the inner shell, and PEG is the outer shell. Again, the thickness of the outer shell, the SLD of the solvent, the incoherent background intensity, and the log-normal size distributions were fixed. The fitting parameters were thus the core radius, the core SLD, the inner shell thickness, the inner shell radius, the outer shell SLD, and an arbitrary pre-factor to account for the non-exact units of intensity. However, despite our success with fitting the data at 30 °C, we were unable to attain a satisfactory fit using reasonable fitting parameters. To ensure that the poor convergence was not a consequence of an incorrect assumption of micelle structure, we repeated the fitting process using an initial guess of a PHPMA core, a PDEGMA inner shell, and a PEG outer shell, yet we were still unable to obtain a satisfactory fit. These fitting challenges suggested that the true micelle structure was likely more complex than the series of concentric spheres assumed by the core-shell-shell form factor. Instead, we believe that the PDEGMA domains may be discrete islands within a PHPMA matrix within the micelle core. To reflect this hypothesis, we thus fit the data to the sum of a spherical form factor and a broad peak form factor, where the broad peak form factor overlaid a Lorentzian peak onto a power law decay. Such a form factor has previously been used to model scattering data from for other soft spherical nanoparticles with multicompartmental cores, *e.g.,* lipid nanoparticles loaded with mRNA.[3, 4] The spherical form factor was intended to capture the overall shape of the micelle, while the broad peak form factor was intended to describe the compartmentalized core. The form factor is provided below:

$$P(q) = [3V\Delta\rho \cdot \frac{\sin(qR) - qRcos(qr)}{qR^3}]^2 + \frac{A}{q^n} + \frac{B}{1 + (|q - q_0|\xi)^m}$$

where V is the sphere volume, R is the sphere radius, A is the Porod law scale factor, n is the Porod exponent, B is the Lorentzian scale factor, q_0 is the broad peak position, ξ is the screening length, and m is the q scaling

exponent. Fitting parameters for our model include: sphere SLD, sphere radius, an arbitrary intensity pre-factor, Porod exponent, Porod scale, Lorentzian scale, Lorentzian exponent, Lorentzian screening length, and peak position. The fit yielded a spherical micelle with a radius of 95 nm with a core that had microphase separated scattering inhomogeneities with a spacing of 30 nm. The extracted fitting parameters are provided below (Supplementary Table 2). Fitted parameters are bolded.

Supplementary Table 2. Extracted Fitting Parameters for at 60 °C SAXS Trace

Parameter	Value
Intensity Pre-factor	**0.03**
Background	0.01
SLD_{PHPMA}	**22.9E-6 A^{-2}**
Core Radius	**18 nm**
SLD_{PDEGMA}	**25.9E-6 A^{-2}**
Inner Shell Thickness	1.5 nm
SLD_{PEG}	**25.8E-6 A^{-2}**
Outer Shell Thickness	4.2 nm
SLD_{H2O}	26.1E-6 A^{-2}
Core Size Distribution	0.3
Shell Size Distribution	0.3
Corona Size Distribution	0.3
**Parameter**	Value
Intensity Pre-factor	**0.1**
Background	0.005
SLD_{sphere}	**25.9E-6 A^{-2}**
Sphere Radius	**95 nm**
SLD_{H2O}	26.1E-6 A^{-2}
Core Size Distribution	0.3
Shell Size Distribution	0.3
Corona Size Distribution	0.3
Porod Scale	**4.62E-8**
Porod Exponent	**4**
Lorentz Scale	**4.32**
Lorentz Screening Length	**5.5 nm**

Peak Position	**0.17 nm^{-1}**
Lorentz Exponent	**4.0**

VII. Supporting Figures

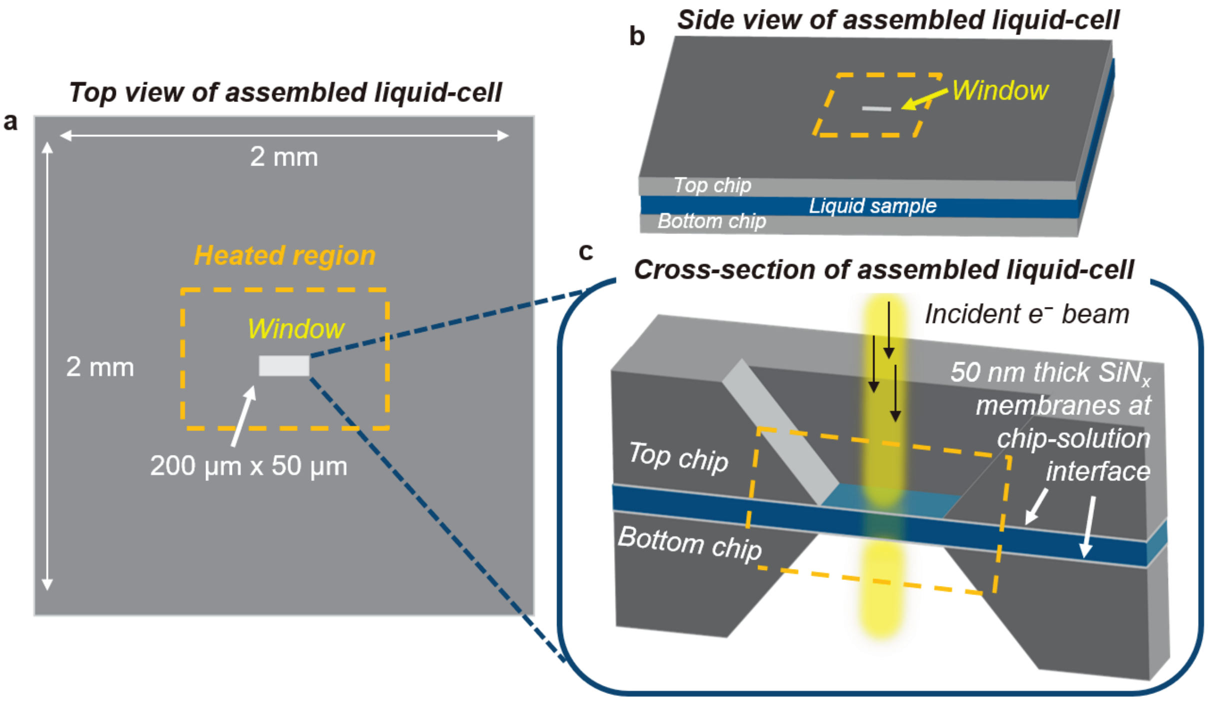

Supplementary Figure 1. Schematic of VT-LCTEM liquid-cell assembly. **a.** Top view of assembled liquid cell. **b.** Side view of assembled liquid cell. **c.** Cross-section of assembled liquid cell.

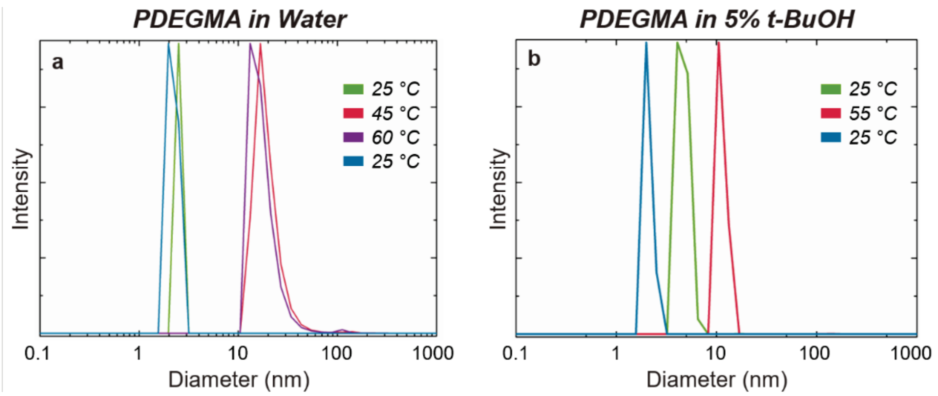

Supplementary Figure 2. VT-DLS of 5 mg mL^{-1} PDEGMA **a.** in water heated from 25 °C (green) to 45 °C (red) to 60 °C (purple) and cooled to 25 °C (blue) and in **b.** 5% t-BuOH heated from 25

°C (green) to 55 °C (red) and cooled to 25 °C (blue). Note the hydrodynamic radius remains constant at and above the cloud point transition.

Damage experiments

Damage experiments on a PDEGMA homopolymer were conducted at 5 mg mL^{-1} at a flux of 2 $e^{-}Å^{-2}s^{-1}$. The first set of experiments were conducted by irradiating each of the four corners of the liquid-cell for 15 minutes, starting at one corner and in turn moving to the next (Supplementary Figure 3b-l). Thus, each corner received a fluence of 1.8 x 10^3 $e^{-}Å^{-2}$, and the total fluence received by the liquid-cell was 7.2 x 10^3 $e^{-}Å^{-2}$. These high fluence conditions led to a destruction of the PDEGMA homopolymer below the detection limit of the MALDI-ToF detector.

Accordingly, the PDEGMA homopolymer was irradiated at the same 2 $e^{-}Å^{-2}s^{-1}$ flux, but for a lower fluence of 600 $e^{-}Å^{-2}$ per corner. These irradiation conditions were employed for only three additives: deuterium oxide, t-butanol, and isopropyl alcohol (Supplementary Figure 4). These additives were chosen because they preserve the LCST transition of the homopolymer and could thus be used in studying the more complex diblock and triblock systems. The deuterium oxide and t-butanol experiments were irradiated for 5 minutes at each of the four corners of the liquid-cell, whereas the isopropyl alcohol experiment was irradiated for 5 minutes at two corners. Thus, the former set experiments received a cumulative fluence of 2.4 x 10^3 and the latter a cumulative fluence of 1.2 x 10^3 $e^{-}Å^{-2}$. A lower total fluence was chosen for the isopropyl alcohol experiment because of the adhesive properties of isopropyl alcohol. The adhesive properties of this scavenger often led to breakage of one or more liquid-cell chips for longer experiments, preventing full MALDI-IMS *post-mortem* analysis (Supplementary Figure 4l).

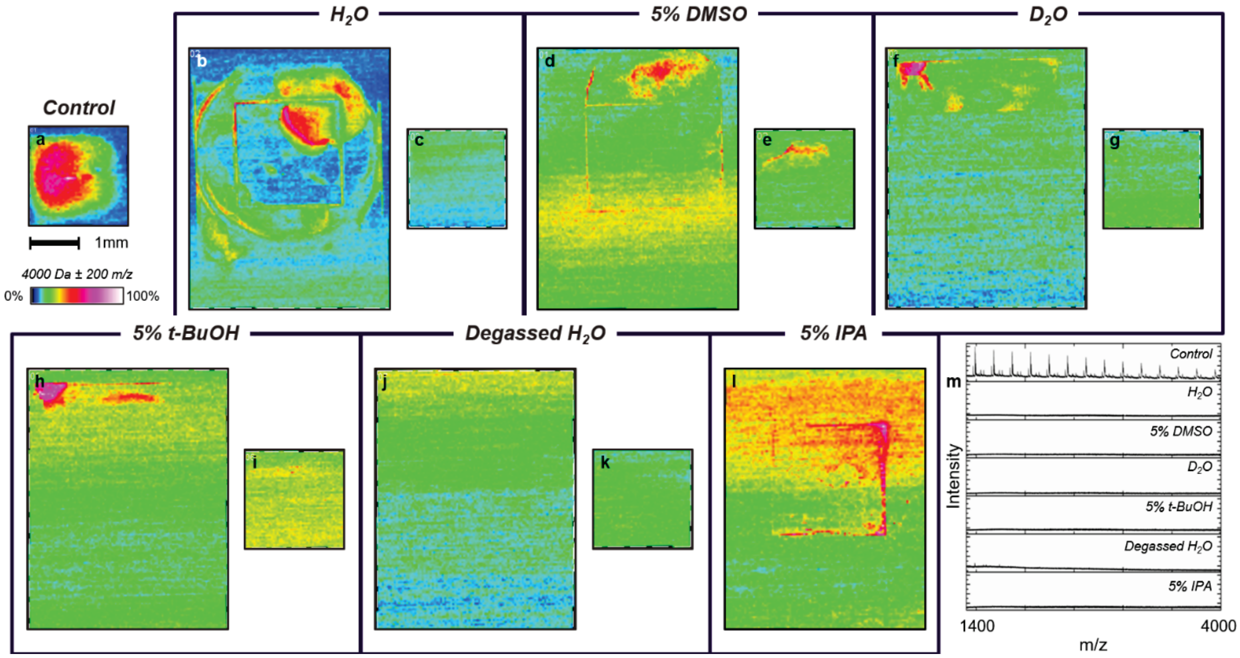

Supplementary Figure 3. MALDI-IMS *post-mortem* analysis of liquid-cell chips following LCTEM experiments for PDEGMA (5 mg mL^{-1}) imaged with different additives at a fixed electron flux of 2 $e^{-}Å^{-2}s^{-1}$ and fluence of 7.2 x 10^3 $e^{-}Å^{-2}$. **a.** Colormap of control, non-irradiated liquid-cell chip coated with 5 mg mL^{-1} PDEGMA. **b-c.** Colormap of top and bottom chips, respectively, of PDEGMA imaged in water, **d-e.** 5% DMSO, **f-g.** D_2O, **h-i.** 5% t-BuOH, and **j-k.** degassed water. **l.** Colormap of top chip of PDEGMA imaged in 5% IPA. Note, bottom chip was broken during chip separation and not measured. **m.** Mass spectra from top to bottom of control, water, 5% DMSO, D_2O, 5% t-BuOH, degassed water, and 5% IPA. All MALDI-IMS colormaps are shown with a mass filter of 4000 ± 200 m/z displayed as 0–100% of total intensity on a logarithmic scale. Note that PDEGMA signal does survive on the chips outside of the imaged region, as shown on the colormap. Note for LCTEM experiment shown in Figure 2f of main text conducted at 20 $e^{-}Å^{-2}s^{-1}$, the liquid-cell dehydrates immediately upon exposure to the electron beam and MALDI-IMS was not measured.

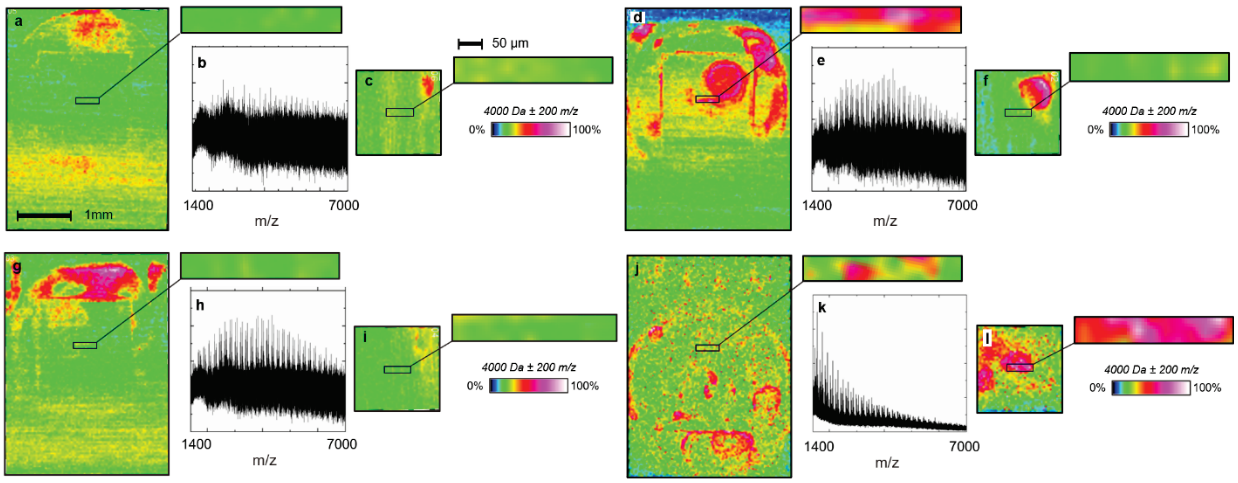

Supplementary Figure 4. MALDI-IMS *post-mortem* analysis of liquid-cell chips following LCTEM experiments for PDEGMA (5 mg mL^{-1}) imaged with different additives at a fixed electron flux of 2 $e^{-}Å^{-2}s^{-1}$ and cumulative fluence of 2.4 x 10^{3} $e^{-}Å^{-2}$ for all but the 5% IPA experiment, which received a cumulative fluence of 1.2 x 10^{3} $e^{-}Å^{-2}$. MALDI-IMS colormap of top chip, mass spectrum, and bottom chip, respectively, for PDEGMA imaged in **a-c.** water, **d-f.** 5% t-BuOH, **g-i.** D_2O, and **j-l.** IPA. The inset shown for each top and bottom chip is the imaged window. All MALDI-IMS colormaps are shown with a mass filter of 4000 ± 200 m/z displayed as 0–100% of total intensity on a logarithmic scale.

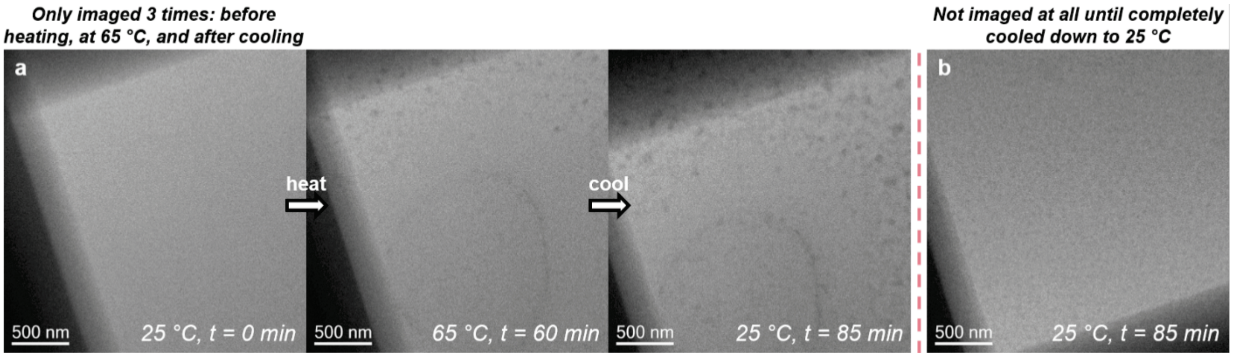

Supplementary Figure 5. Control VT-LCTEM experiments on 10 mg mL^{-1} PEG-b-PDEGMA in water imaged at a flux of 0.46 $e^{-}Å^{-2}s^{-1}$. **a.** Single frames of VT-LCTEM experiment on diblock heated to 65 °C, where the sample was only imaged before heating, at 65 °C, and upon cooling. **b.** Single frame of VT-LCTEM experiment on diblock only imaged upon cooling to 25 °C.

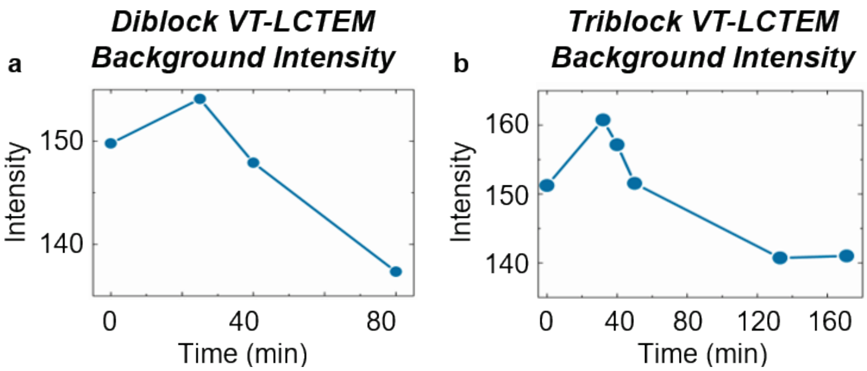

Supplementary Figure 6. Average liquid background intensity measured for VT-LCTEM experiments on **a.** PEG-b-PDEGMA diblock copolymer shown in Figure 3 and **b.** PEG-b-PDEGMA-b-PHPMA triblock copolymer shown in Figure 4.

Description of LCTEM Experiments with Alcohol Cosolvents

Given the persistence of the cloud point in alcohol mixtures and the ability of alcohols to scavenge destructive •OH radicals, we also attempted to visualize assembly formation in the presence of IPA. In the bulk, PEG-b-PDEGMA assembly formation upon heating occurs with up to 30% IPA, but assembly formation under these solvent conditions was suppressed in the liquid-cell, likely due to concentration gradients in the thin liquid layer (Supplementary Figure 22). However, with 15% IPA, assembly formation in the PEG-b-PDEGMA diblock copolymer was found to occur, though somewhat nonuniformly due to heterogeneities in the IPA distribution in the liquid-cell (Supplementary Figure 7). We also attempted to visualize assembly formation in 5% t-BuOH. Though assembly formation still occurs in the bulk for up to 10% t-BuOH, in over five VT-LCTEM experiments using only 5% t-BuOH, we saw weak evidence of assembly formation in only one experiment (Supplementary Figure 22). Given the inherently thin liquid layer required for LCTEM analysis, the failure of the LCST transition in the presence of t-BuOH may be due the existence of t-BuOH concentration gradients, with areas of high and low local t-BuOH concentrations, that prevent effective polymer-water interactions critical to the cloud point transition. The presence of concentration gradients in liquid-cell experiments is likely a

manifestation of the high surface area of the liquid-cell windows compared to bulk conditions.[5, 6] Additionally, slight variations in the solution concentration during loading the low sample volumes required for LCTEM could also play a role in the formation of concentration gradients.

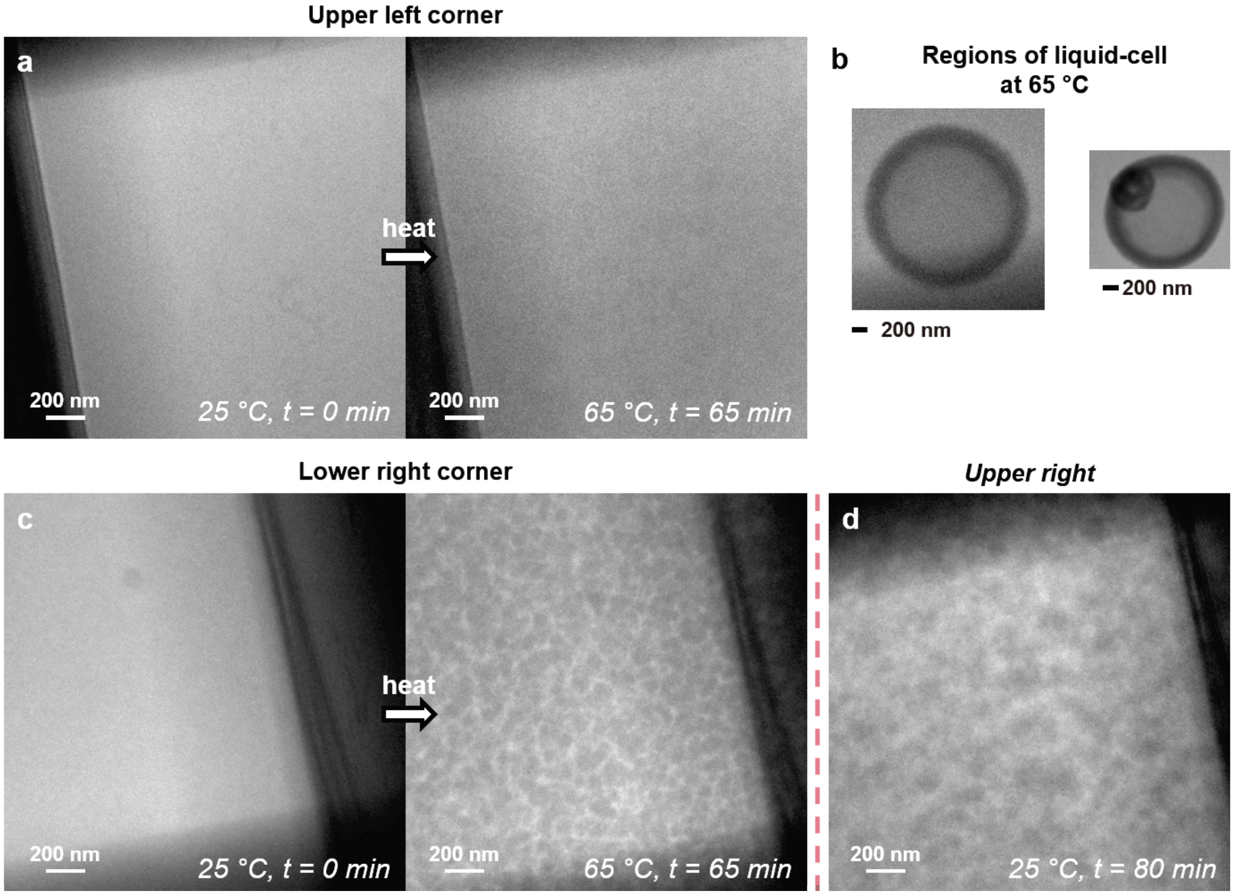

Supplementary Figure 7. Single frames of VT-LCTEM experiment 10 mg mL^{-1} PEG-b-PDEGMA in 15% IPA imaged at a flux of 0.5 $e^{-}Å^{-2}s^{-1}$ and heated to 65 °C. **a.** Single frames of upper left corner with no evidence of assembly. **b.** Regions in liquid-cell at 65 °C, highlighting assembly formation. **c.** Single frames of lower right control corner, imaged only before heating and at 65 °C. **d.** Single frame of upper right control corner, imaged only at completion of heating-cooling cycle.

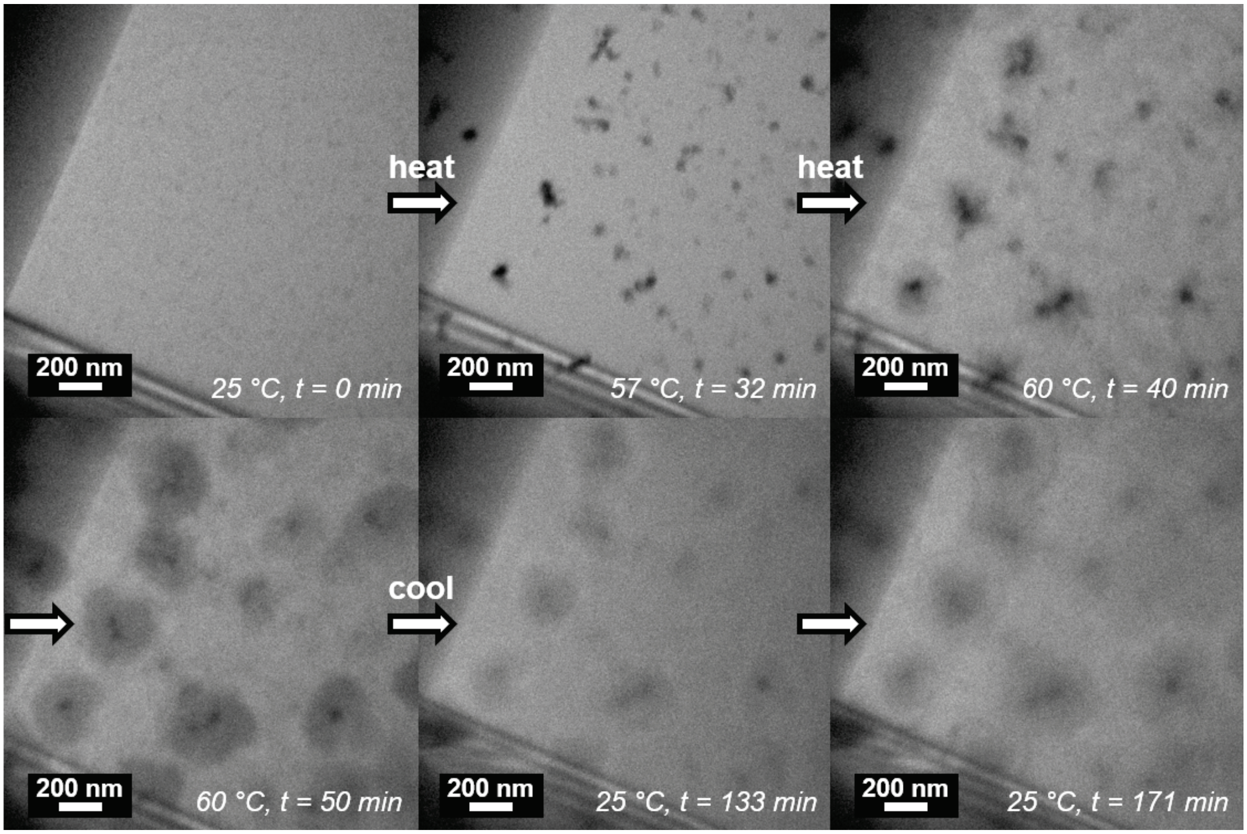

Supplementary Figure 8. Single frames of VT-LCTEM experiment on 15 wt% triblock in water imaged at a flux of 0.8 e^{-} $Å^{-2}$ s and heated to 60 °C. A cropped region of interest from these frames was used for the analysis conducted in Figure 4.

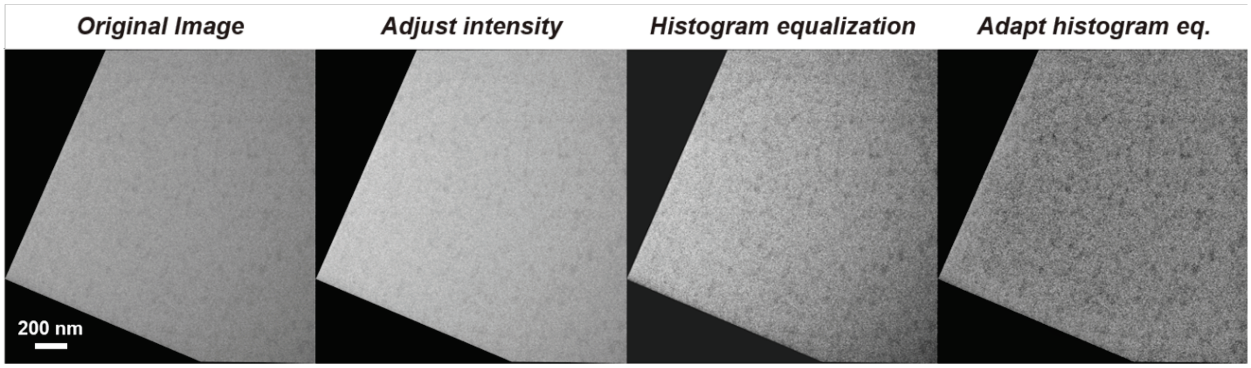

Supplementary Figure 9. Image adjustments performed in MATLAB for room temperature morphology observed in VT-LCTEM experiment on 15 wt% PEG-b-PDEGMA-b-PHPMA in water imaged at a flux of 0.8 $e^{-}Å^{-2}s^{-1}$. From left to right, the original image, image enhanced with imadjust, image enhanced with histeq, and image enhanced with adapthisteq.

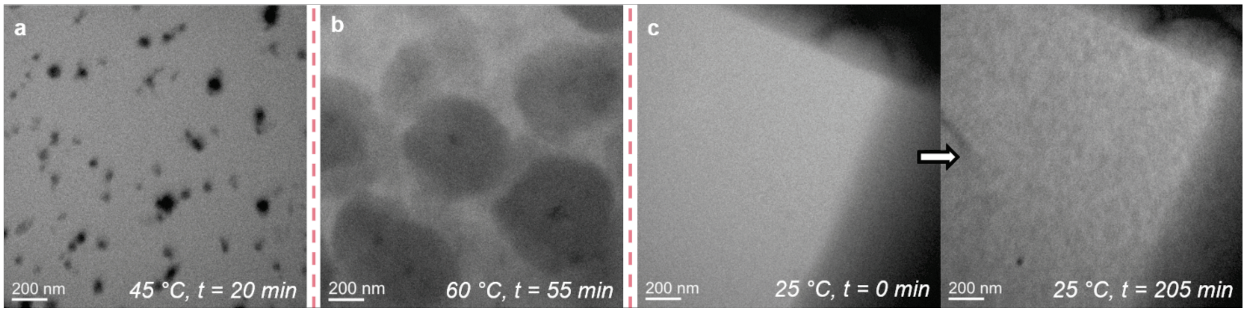

Supplementary Figure 10. Single frames of VT-LCTEM experiment on 15 wt% PEG-b-PDEGMA-b-PHPMA in water imaged at a flux of 0.8 $e^{-}Å^{-2}s^{-1}$ and heated to 60 °C. **a.** Single frame of region of liquid-cell imaged for the first time at 45°C. **b.** Single frame of region of liquid-cell imaged for the first time at 60°C. **c.** Single frames of control VT-LCTEM experiment of triblock only imaged before heating and then upon cooling to 25 °C, highlighting the irreversibility of the triblock transformation in the absence of the electron beam.

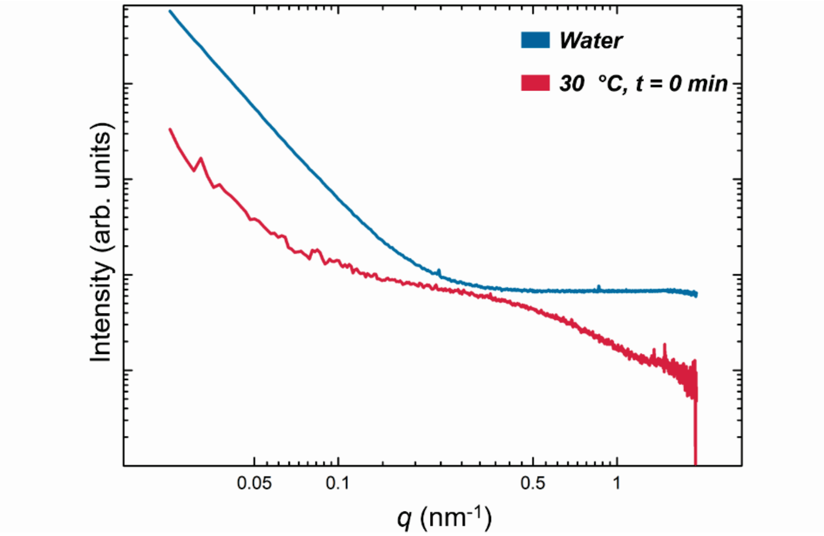

Supplementary Figure 11. VT-SAXS data of 30 °C trace overlaid with water trace.

Description of PEG-b-PDMAEMA System

With the PEG-b-PDEGMA diblock system, we demonstrate the first example of a thermoresponsive diblock copolymer assembly being directly observed in solution upon heating to temperature. Notably, our approach for studying thermoresponsive diblock copolymers is generalizable, and we were also able to visualize the elevated temperature morphology of PEG-b-poly(2-(dimethylamino)ethyl methacrylate) (PDMAEMA), which likewise contains a

thermoresponsive PDMAEMA block (Supplementary Figures 12 and 29). Note that we were not able to observe nanostructure-assembly in any system we studied with polymer concentrations less than 10 mg mL^{-1}, as we have found that a high local polymer concentration is required in the diffusion-limited, liquid-cell environment (Supplementary Figure 13).

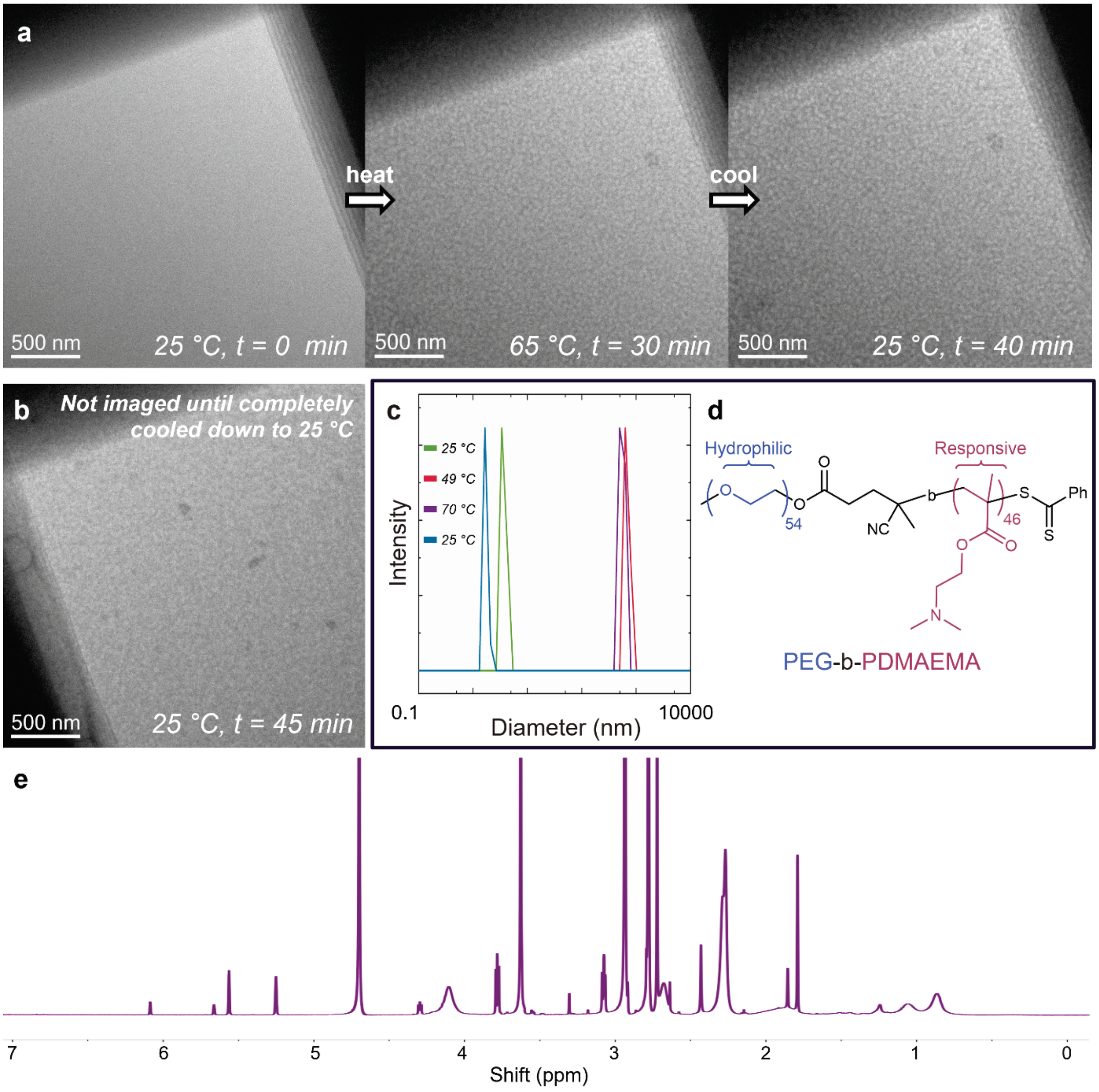

Supplementary Figure 12. VT-LCTEM experiments on 10 mg mL^{-1} PEG-b-PDMAEMA in water imaged at a flux of 0.46 e^{-}Å^{-2}s^{-1}. **a.** Single frames of VT-LCTEM experiment on diblock heated to 65 °C and cooled to 25°C. **b.** Single frame of control VT-LCTEM experiment on diblock only imaged upon cooling to 25 °C. **c.** VT-DLS performed on 5 mg mL^{-1} diblock in water, heated

from 25 °C (green) to 49 °C (red) to 70 °C (purple) and cooled to 25 °C (blue). **d.** Structure of diblock. **e.** Crude ^{1}H NMR spectrum from synthesis of PEG-b-PDMAEMA measured in deuterium oxide.

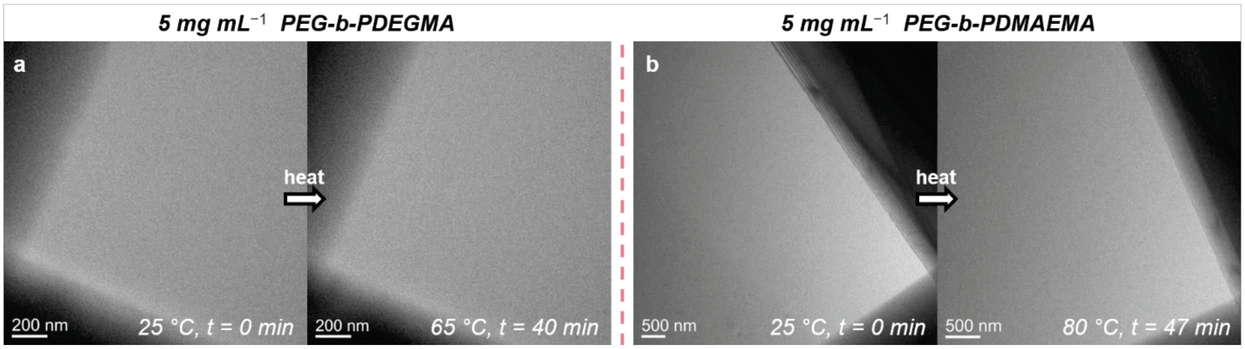

Supplementary Figure 13. VT-LCTEM control experiments on thermoresponsive diblocks imaged in water. **a.** Single frames of VT-LCTEM experiment on 5 mg mL^{-1} PEG-b-PDEGMA imaged at a flux of 0.8 $e^{-}Å^{-2}s^{-1}$ and heated to 65 °C. **b.** Single frames of VT-LCTEM experiment on 5 mg mL^{-1} PEG-b-PDMAEMA imaged at a flux of 0.46 $e^{-}Å^{-2}s^{-1}$ and heated to 80 °C. Note the lack of observable assembly in both cases under the dilute conditions used.

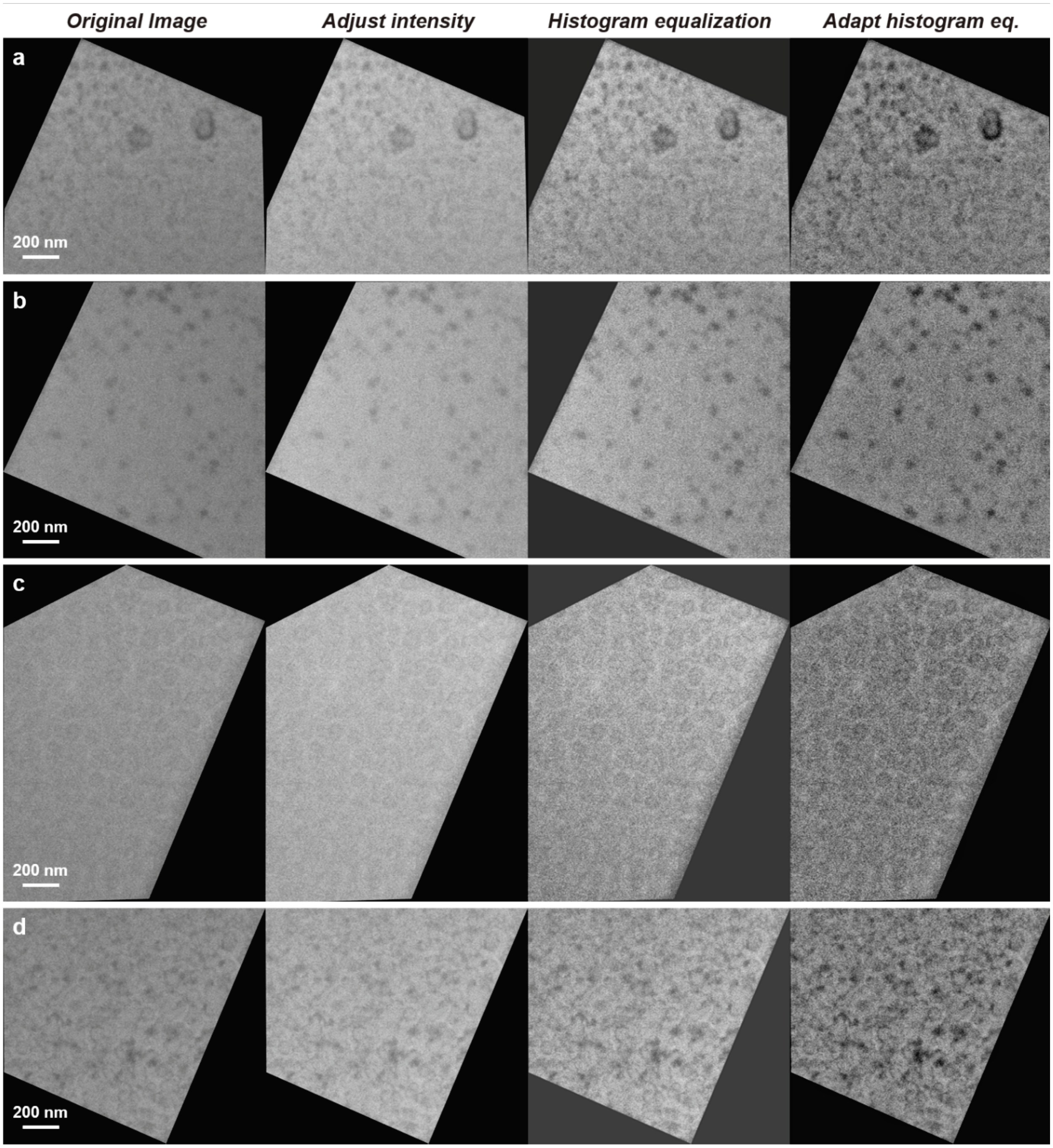

Supplementary Figure 14. Image adjustments performed in MATLAB for elevated temperature morphology (50 °C) observed in four VT-LCTEM experiments on 10 mg mL^{-1} PEG-b-PDEGMA in water imaged at a flux of 0.8 $e^{-}Å^{-2}s^{-1}$. **a-d.** From left to right, the original image, image enhanced with imadjust, image enhanced with histeq, and image enhanced with adapthisteq.

Polymer-Solvent Interactions During VT-LCTEM Experiments

In several VT-LCTEM experiments, we observed liquid thinning or exclusion from the liquid-cell window upon heating (Supplementary Figures 15, 19, 20). As the LCST transition is a manifestation of polymer-solvent interactions, changes in liquid thickness under LCTEM conditions are not entirely surprising. Notably, in two experiments with PEG-b-PDEGMA, liquid thinning upon heating was observed upon the phase separation of the PDEGMA block (Supplementary Figures 12 and 19). Likewise, the PDEGMA homopolymer exhibited a clear phase separation upon overnight heating, featuring a dense polymeric phase and a water phase (Supplementary Figure 20).

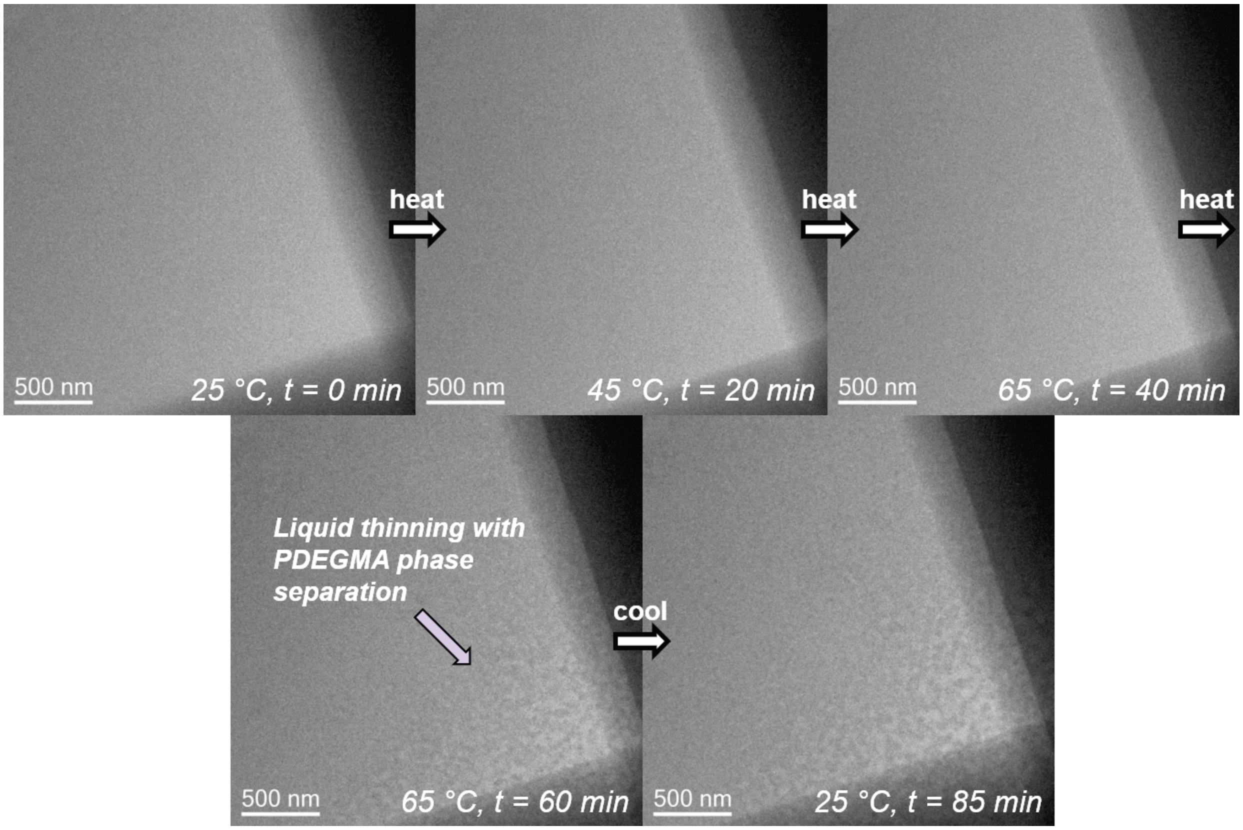

Supplementary Figure 15. Single frames of VT-LCTEM experiment on 10 mg mL^{-1} PEG-b-PDEGMA in water imaged at a flux of 0.46 e^{-}Å^{-2}s^{-1} and heated to 65 °C. Note the apparent liquid thinning upon the cloud point transition of the PDEGMA block due to the exclusion of water from the polymer.

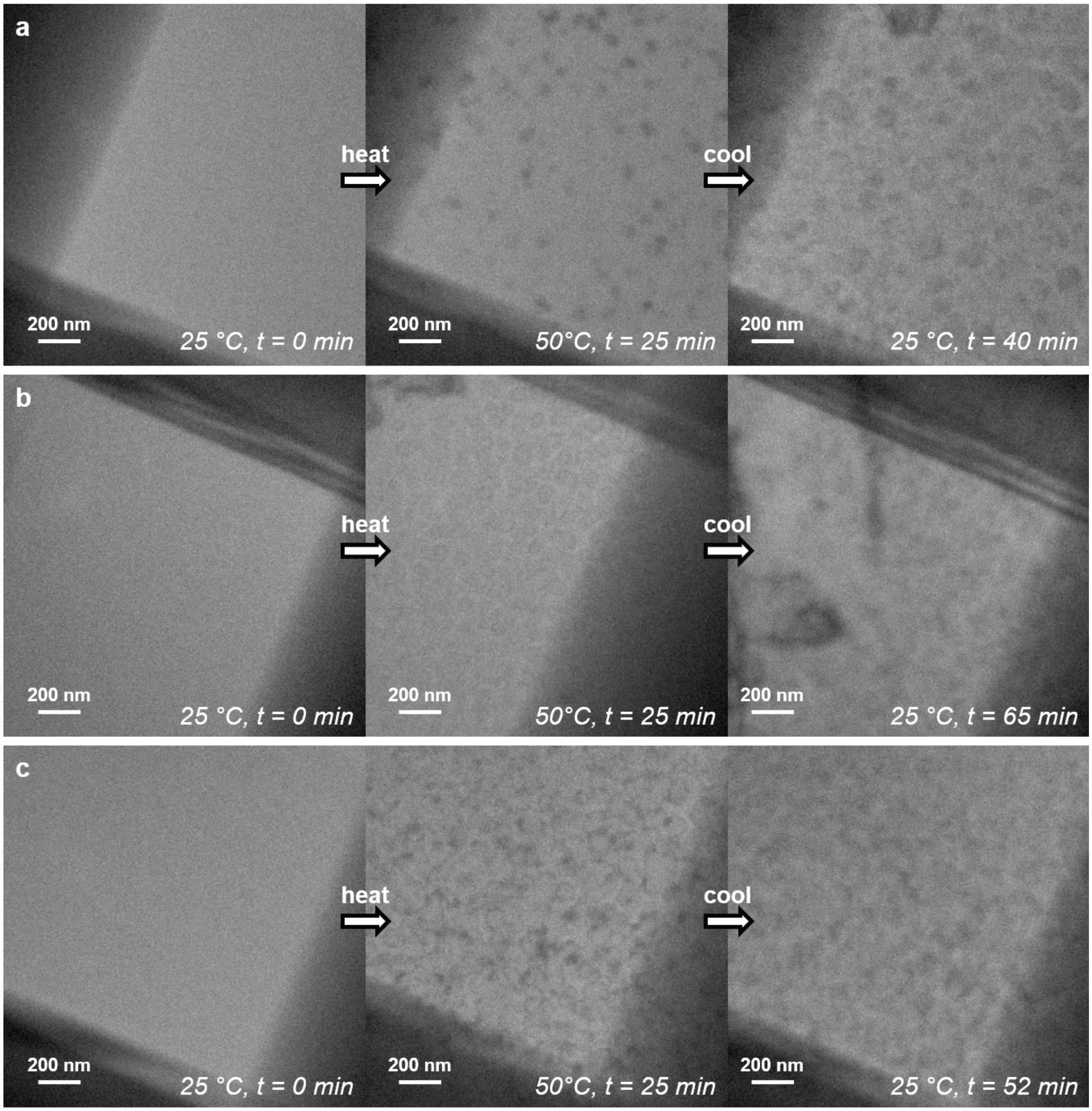

Supplementary Figure 16. Repeated VT-LCTEM experiments on 10 mg mL^{-1} PEG-b-PDEGMA in water imaged at a flux of 0.8 $e^{-}Å^{-2}s^{-1}$. **a-c** Single frames of control VT-LCTEM experiment on diblock heated to 50 °C and cooled back to room temperature. Note the distinct morphology observed in each experiment, highlighting the role of liquid thickness and potential concentration gradients.

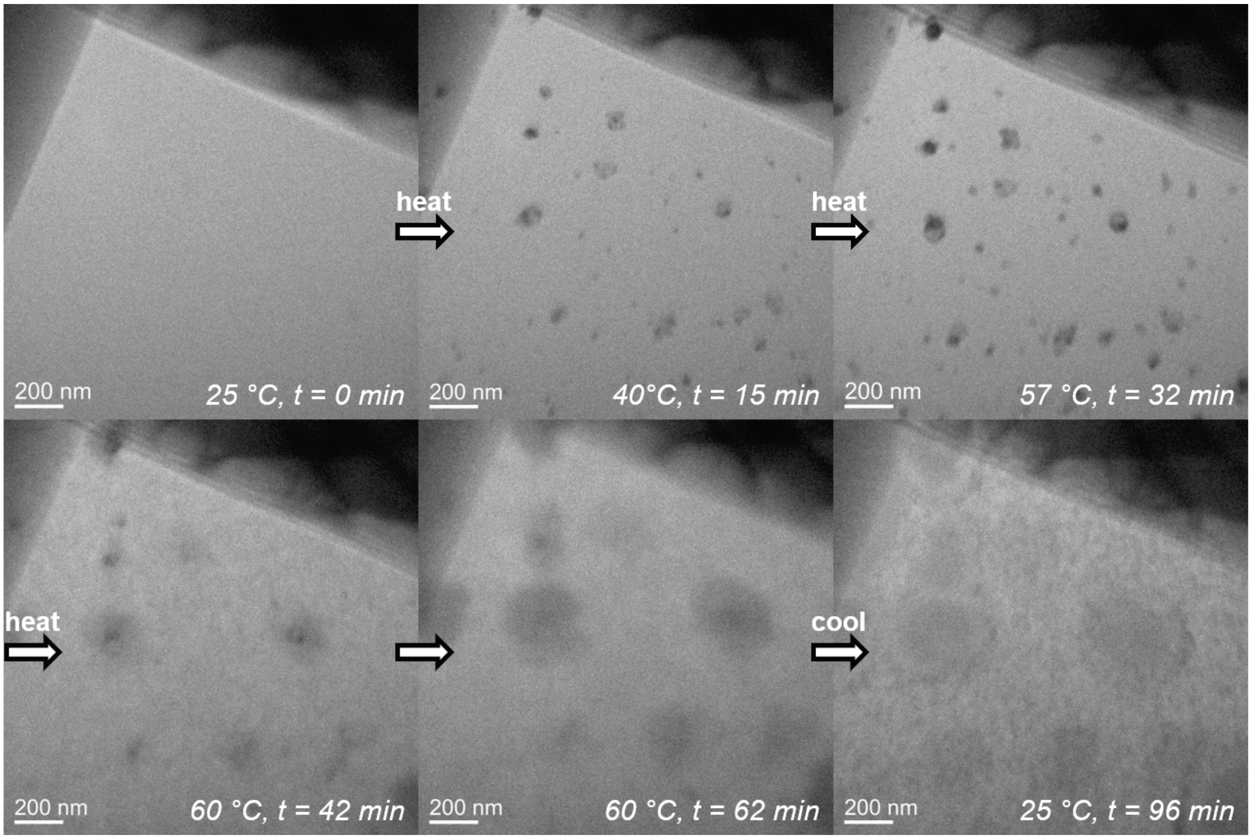

Supplementary Figure 17. Single frames of VT-LCTEM experiment on 15 wt% PEG-b-PDEGMA-b-PHPMA in water imaged at a flux of 0.8 $e^{-}Å^{-2}s^{-1}$ and heated to 60 °C.

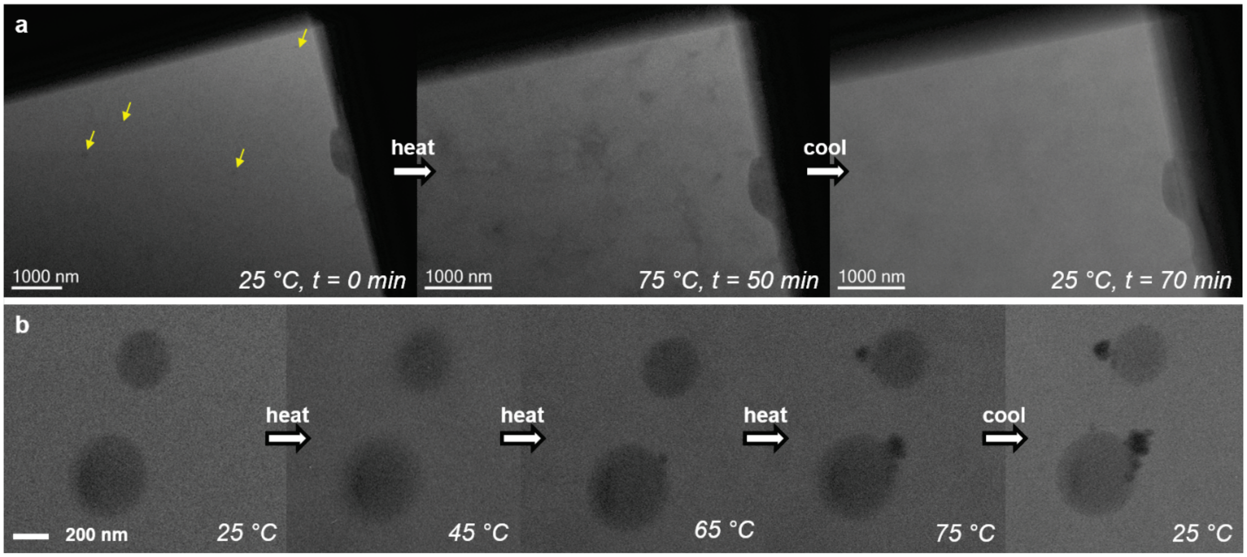

Supplementary Figure 18. Single frames of VT-LCTEM experiment on 15 wt% PEG-b-PDEGMA-b-PHPMA in water imaged at a flux of 0.1 $e^{-}Å^{-2}s^{-1}$ and heated to 75 °C. **a.** Single frames of VT-LCTEM experiment at corner of liquid-cell where liquid layer is thinnest. **b.** Region of interest near center of liquid-cell where liquid layer is thicker.

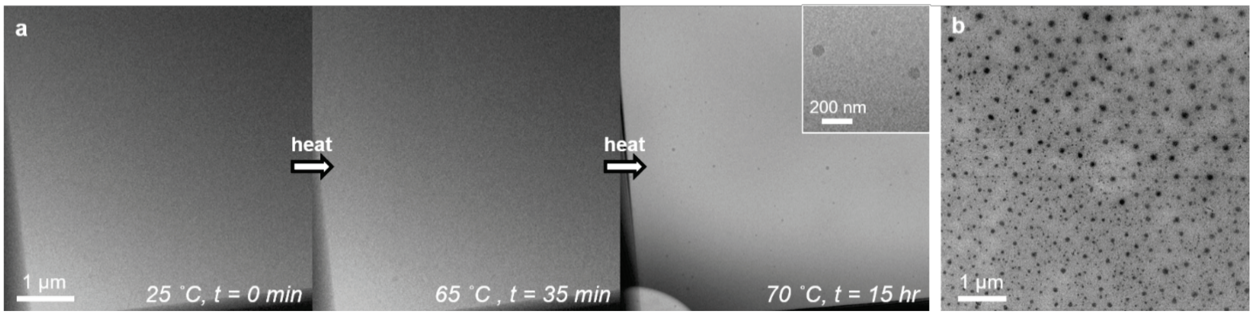

Supplementary Figure 19. Single frames of VT-LCTEM experiment on 10 mg mL^{-1} PEG-b-PDEGMA in water imaged at a flux of 0.1 $e^{-}Å^{-2}s^{-1}$. **a.** Single frames of liquid-cell before heating followed by heating to 65 °C and then to 70 °C overnight. Note liquid-thinning in the third frame makes the formation of assemblies more easily distinguishable. **b.** *Post-mortem* dry state TEM of dry SiNx chips, showing uniform formation of spherical assemblies all over the chips.

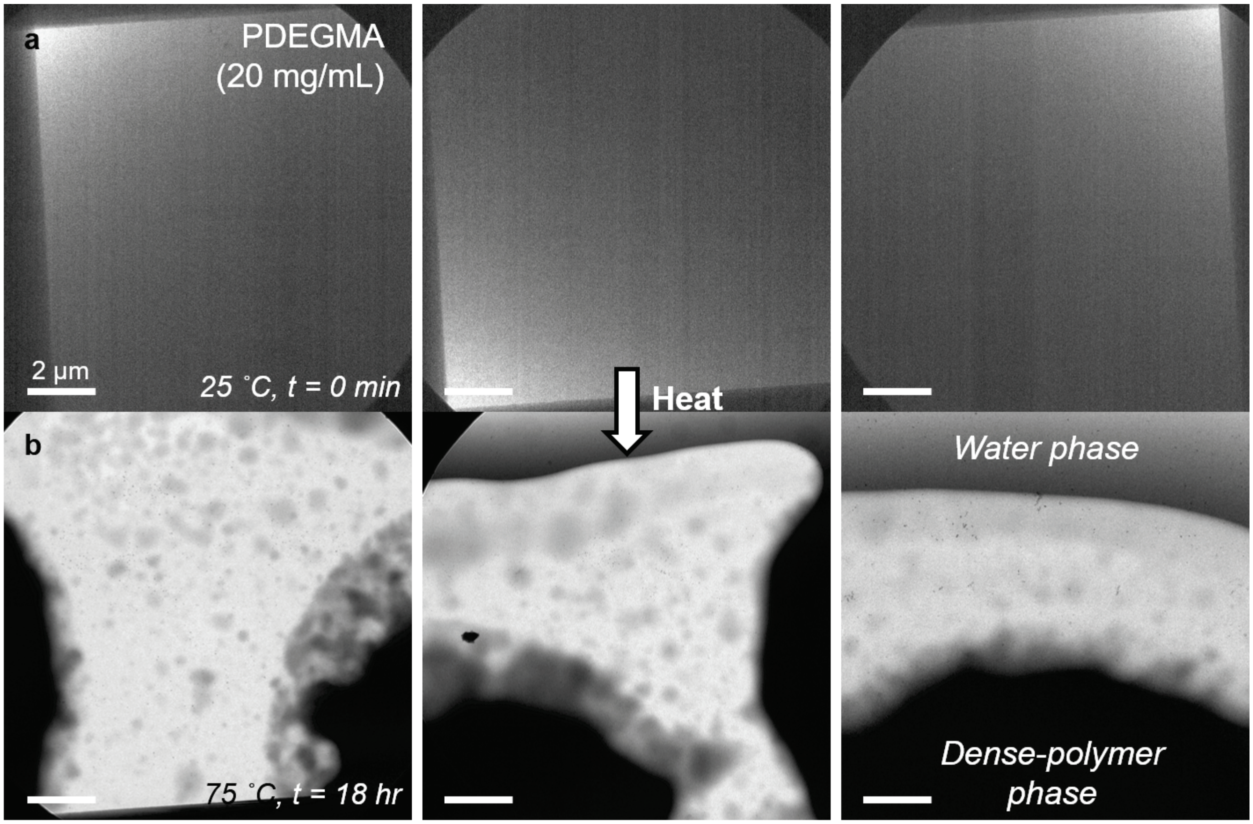

Supplementary Figure 20. Single frames of VT-LCTEM experiment on 20 mg mL^{-1} PDEGMA in water imaged at a flux of 0.11 e^{-}Å^{-2}s^{-1} and heated to 75 °C. **a.** Three corners of liquid-cell before heating, highlighting the absence of structures. **b.** Same three corners after being held at 75 °C for 18 hours. Note the phase separation of the polymer and solvent as triggered by the LCST transition.

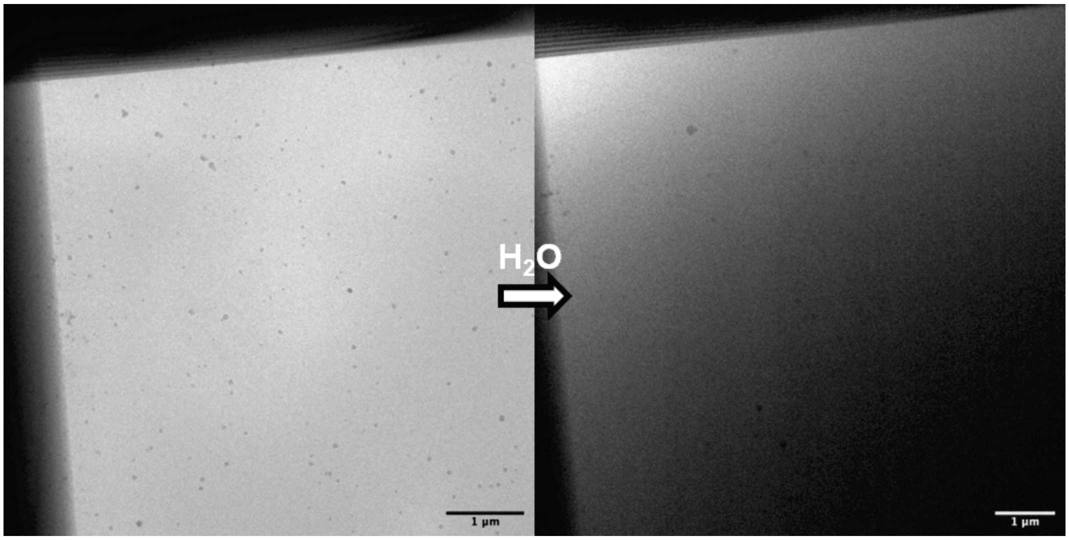

Supplementary Figure 21. Liquid-cell with triblock nanoassemblies before and after flowing in water imaged at a flux of 0.1 e^{-}Å^{-2}s^{-1} at room temperature. The triblock assemblies were purposely dried and rehydrated to highlight the difference in contrast.

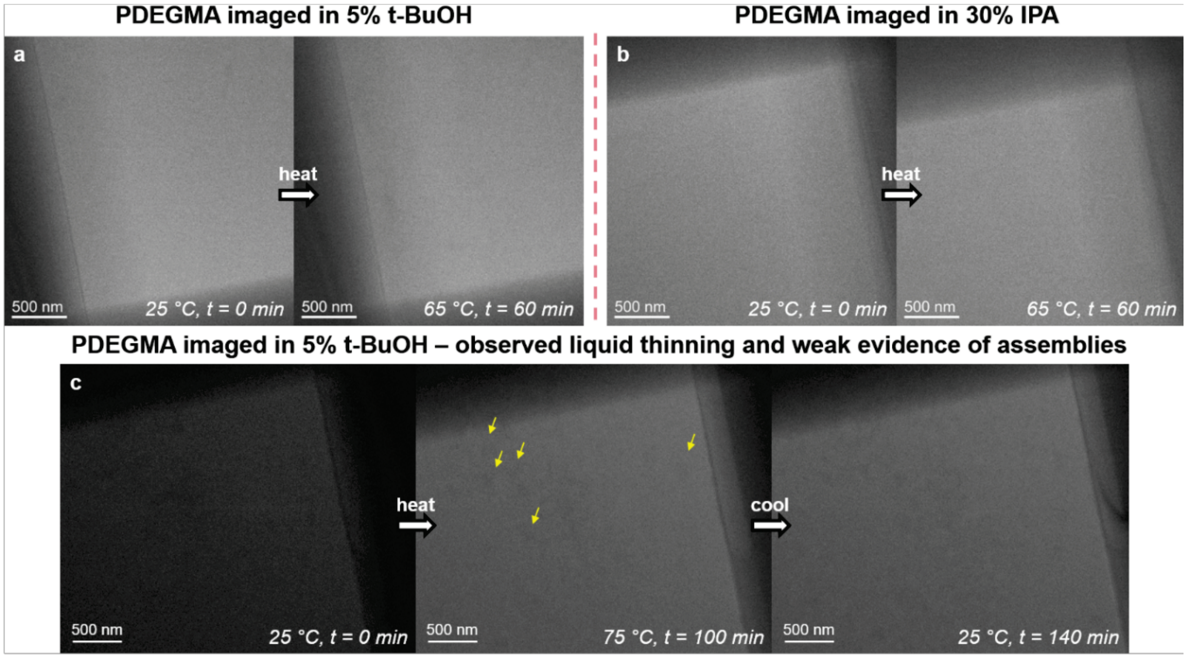

Supplementary Figure 22. Single frames of VT-LCTEM experiment 10 mg mL^{-1} PEG-b-PDEGMA in water-alcohol mixtures. **a.** Single frames of PEG-b-PDEGMA imaged in 5% t-BuOH showing no clear evidence of assembly, imaged at a flux of 0.5 e^{-}Å^{-2}s^{-1} and heated to 65 °C. **b.** Single frames of PEG-b-PDEGMA imaged in 30% IPA showing no clear evidence of assembly, imaged at a flux of 0.5 e^{-}Å^{-2}s^{-1} and heated to 65 °C. **c.** Single frames of PEG-b-PDEGMA imaged

in 5% t-BuOH showing slight evidence of assembly, imaged at a flux of 0.3 $e^{-}Å^{-2}s^{-1}$ and heated to 75 °C.

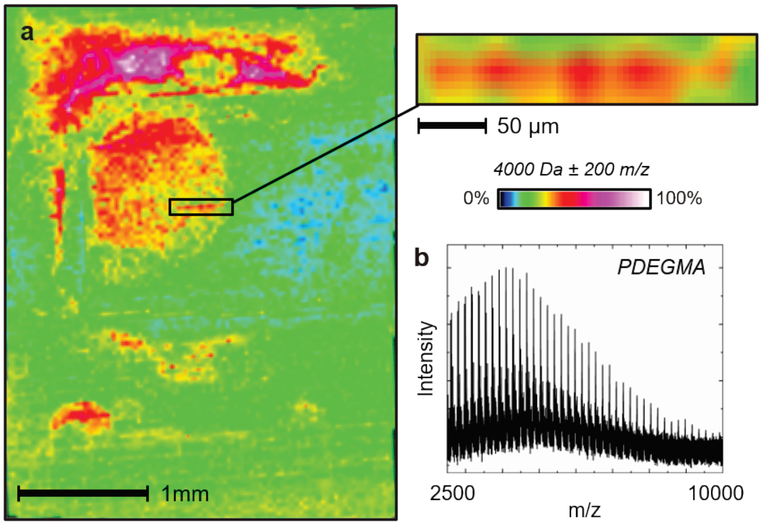

Supplementary Figure 23. Additional MALDI-IMS data for experiment shown in Figure 3. **a.** MALDI-IMS colormap of top chip with inset showing imaged window from VT-LCTEM experiment on PDEGMA shown with a mass filter of 4000 ± 200 m/z displayed as 0–100% of total intensity on a logarithmic scale.

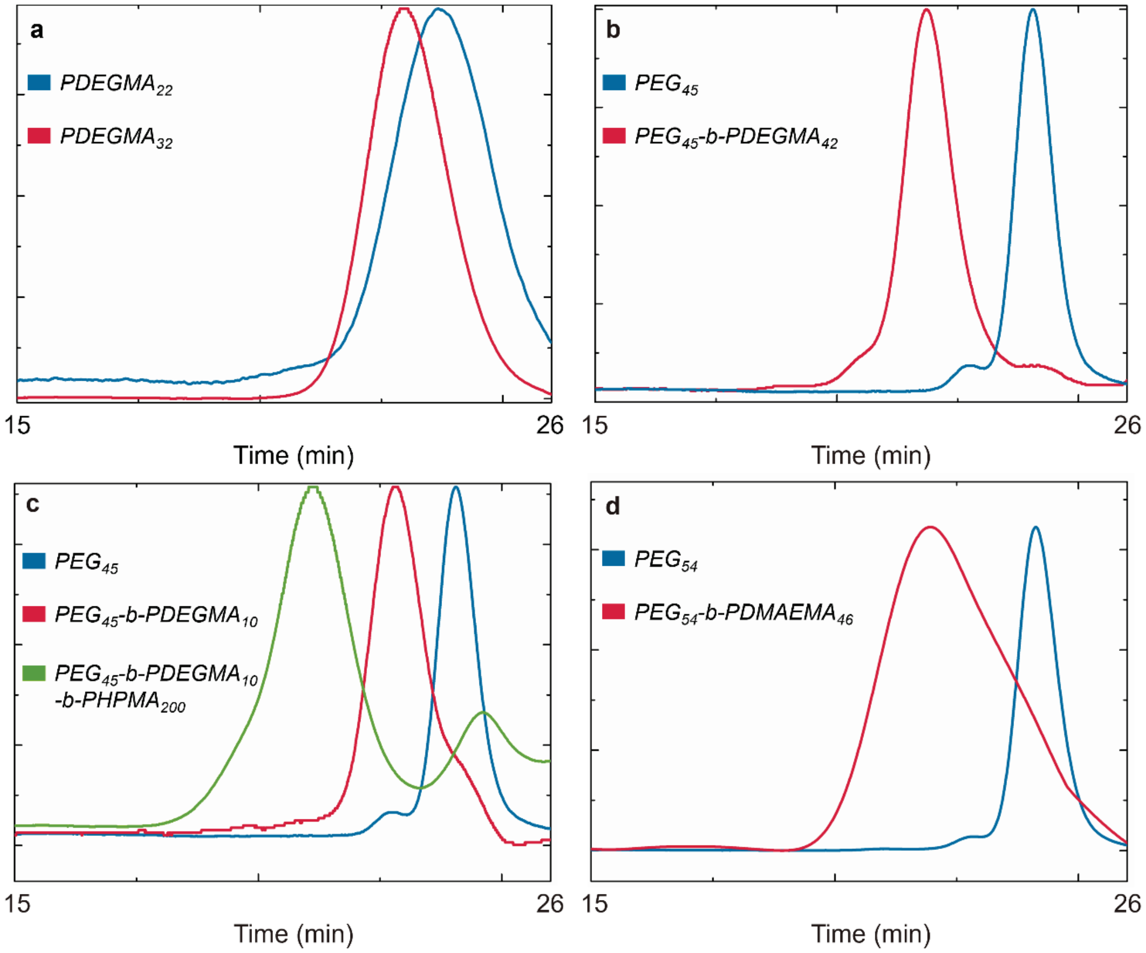

e	Polymer	Polydispersity
	$PDEGMA_{22}$	1.16
	$PDEGMA_{32}$	1.09
	PEG_{45}	1.05
	PEG_{45}-b-$PDEGMA_{42}$	1.06
	PEG_{45}-b-$PDEGMA_{10}$	1.10
	PEG_{45}-b-$PDEGMA_{10}$-b-$PHPMA_{200}$	1.14
	PEG_{54}	1.01
	PEG_{54}-b-$PDMAEMA_{46}$	1.20

Supplementary Figure 24. SEC-MALS traces for block copolymers synthesized via RAFT. SEC-MALS traces for **a.** PDEGMA homopolymers used for LCTEM studies, **b.** commercially available 2 kDa PEG macro-CTA and PEG-b-PDEGMA copolymer made by chain extension of the macro-CTA, **c.** 2 kDa PEG macro-CTA, PEG-b-PDEGMA diblock made by chain extension of the macro-CTA, and triblock copolymer made by chain extension of the diblock, and **d.** commercially available 2.4 kDa PEG macro-CTA, PEG-b-PDMAEMA diblock made by chain extension of the macro-CTA. **e.** Polydispersity index for each polymer as measured by SEC-MALS.

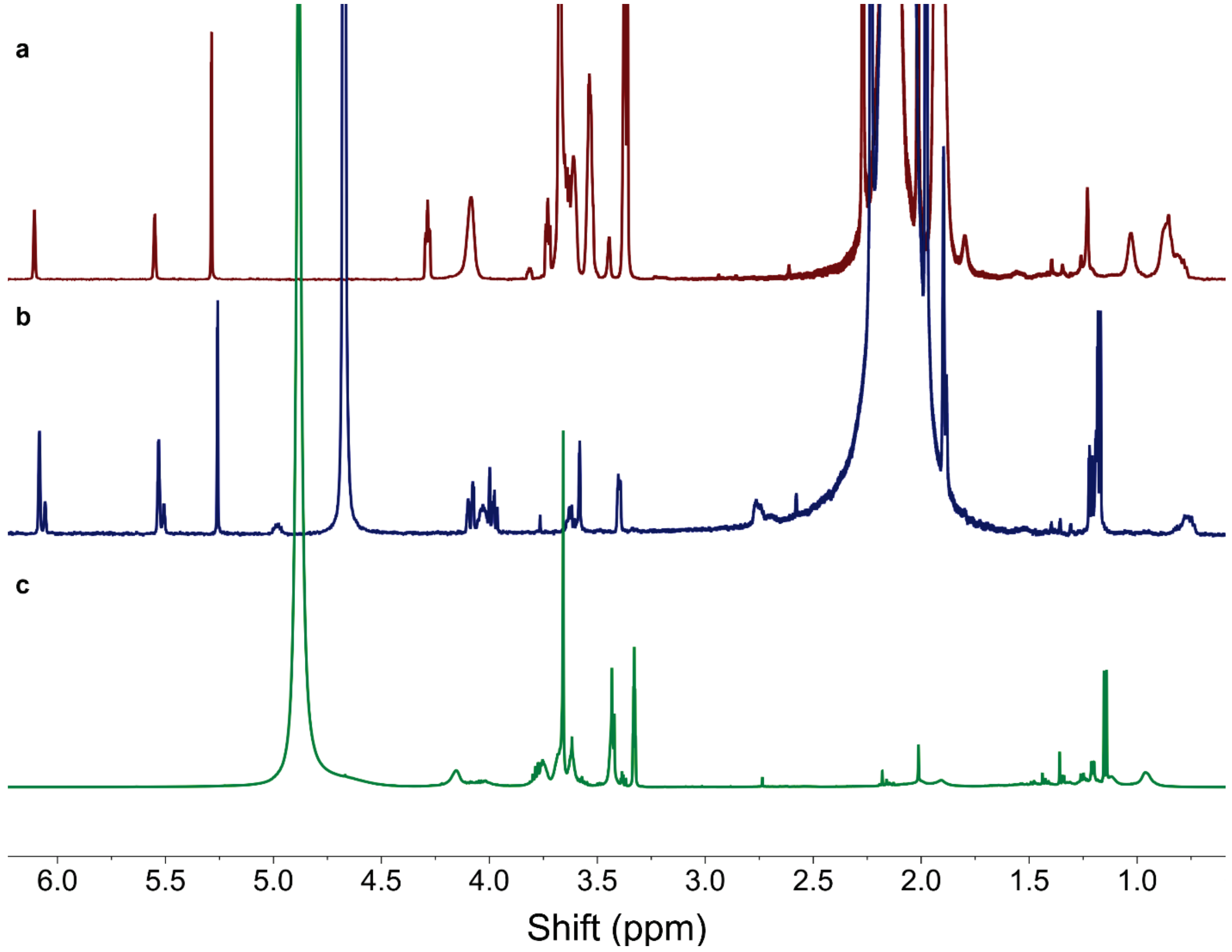

Supplementary Figure 25. Crude ^{1}H NMR spectra from syntheses of **a.** PDEGMA measured in deuterated chloroform, **b.** PEG-b-PDEGMA measured in deuterated chloroform, and **c.** PEG-b-PDEGMA-b-PHPMA measured in deuterated methanol.

VII. Synthesis

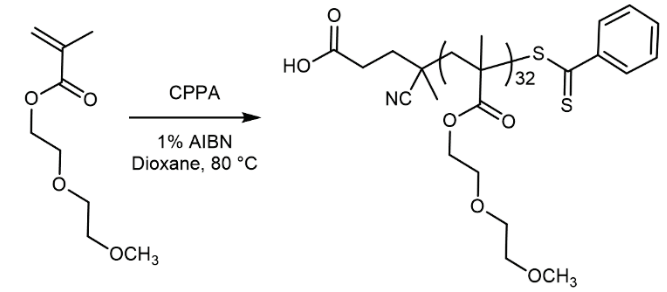

Supplementary Figure 26. Synthesis of PDEGMA homopolymer.

Synthesis of PDEGMA To a 20 mL glass vial equipped with a septum, di(ethylene glycol) methyl ether methacrylate (DEGMA, 500 mg, 0.27 mmol), azobisisobutyronitrile (AIBN, 0.087 mg, 0.53 μmol), 4-cyano-4-[(dodecylsulfanylthiocarbonyl)sulfanyl]pentanoic acid (CDPA, 21.4 mg, 0.05 mmol), and dioxane (1 mL) were added (Scheme 1). N_2 gas was bubbled through the solution for 1 hour before the vial was heated in an oil bath to 80 °C for 160 minutes under stirring. The reaction was quenched by submerging the reaction mixture in liquid nitrogen. The crude product was precipitated into hexanes and dried under vacuum. ^{1}H NMR was used to evaluate the conversion, as discussed in previous studies (Supplementary Figure 25a).[7, 8, 9]

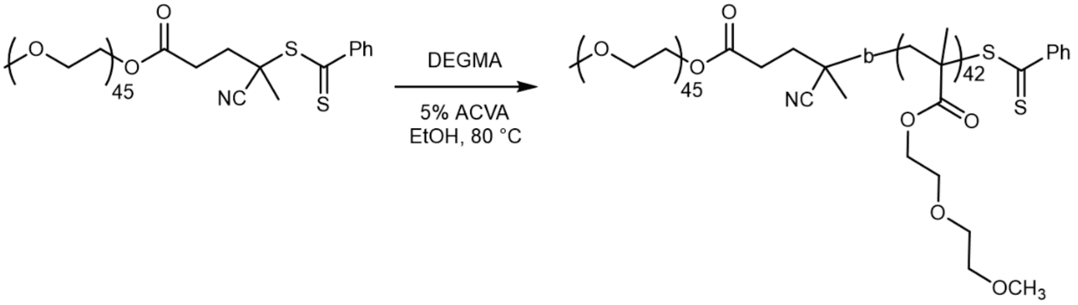

Supplementary Figure 27. Synthesis of PEG-b-PDEGMA diblock copolymer.

Synthesis of PEG-b-PDEGMA To a 20 mL glass vial equipped with a septum, 2 kDa poly(ethylene glycol) 4-cyano-4- (phenylcarbonothioylthio)pentanoate (PEG, 56.3 mg, 0.03 mmol), 4,4′-azobis(4-cyanovaleric acid) (ACVA, 0.4 mg, 0.32 μmol), HPMA (97.0 mg, 0.67 mmol), and milli-Q water (550 μL) were added (Scheme 2). N_2 gas was bubbled through the solution for 2 hours before the vial was heated in an oil bath to 80 °C for 19 hours under stirring. The reaction was quenched by submerging the reaction mixture in ice water. ^{1}H NMR was used to evaluate the conversion, as discussed in previous studies (Supplementary Figure 25b).[7, 8, 9]

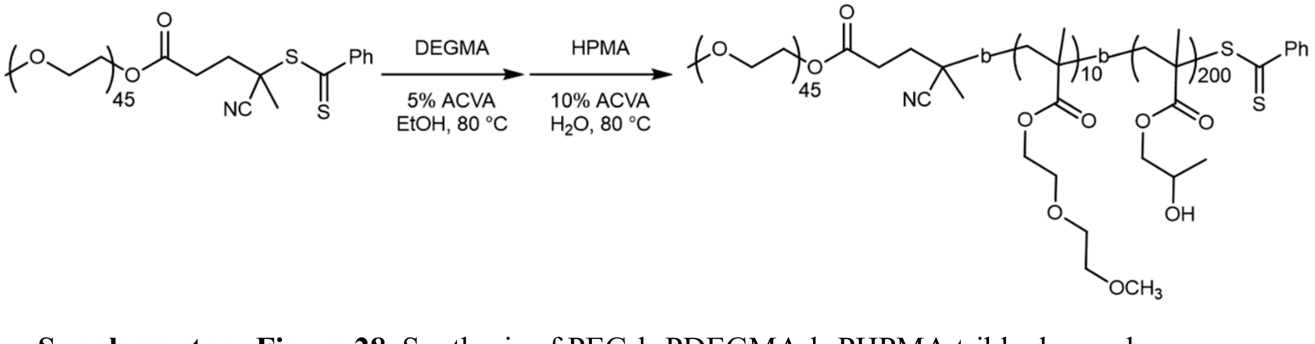

Supplementary Figure 28. Synthesis of PEG-b-PDEGMA-b-PHPMA triblock copolymer.

Synthesis of PEG-b-PDEGMA-b-PHPMA To a 20 mL glass vial equipped with a septum, PEG_{45}-b-$PDEGMA_{10}$ (57.5 mg, 0.02 mmol), ACVA (0.09 mg, 1.43 μmol), DEGMA (265 mg, 1.41 mmol), and anhydrous ethanol (106 μL) were added (Scheme 3). N_2 gas was bubbled through the solution for 1 hour before the vial was heated in an oil bath to 80 °C for 3 hours under stirring. The reaction was quenched by submerging the reaction mixture in liquid nitrogen. The crude product was precipitated into hexanes and dried under vacuum. ^{1}H NMR was used to evaluate the conversion, as discussed in previous studies (Supplementary Figure 25c).[10]

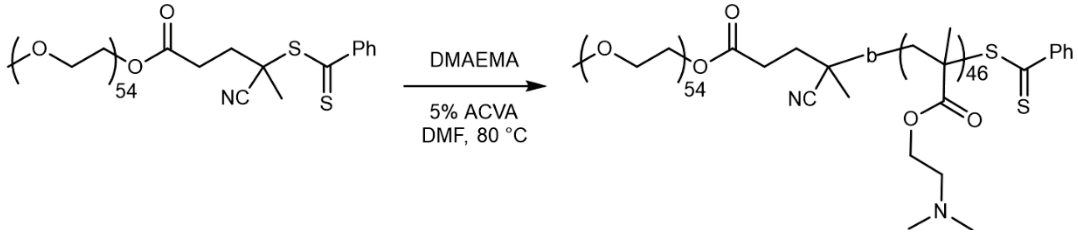

Supplementary Figure 29. Synthesis of PEG-b-PDMAEMA diblock copolymer.

Synthesis of PEG-b-PDMAEMA To a 20 mL glass vial equipped with a septum, 2.4 kDa poly(ethylene glycol) methyl ether (4-cyano-4-pentanoate dodecyl trithiocarbonate) (155 mg, 0.065 mmol), ACVA (0.91 mg, 3.2 μmol), 2-(dimethylamino)ethyl methacrylate (DMAEMA, 509 mg, 3.23 mmol), and dimethylformamide (1.30 mL) were added (Scheme 4). N_2 gas was bubbled through the solution for 1 hours before the vial was heated in an oil bath to 80 °C for 21 hours under stirring. The reaction was quenched by submerging the reaction mixture in liquid nitrogen. ^{1}H NMR was used to evaluate the conversion, as discussed in previous studies (Supplementary Figure 12e).[7]

VIII. Kinetics Experiments

Kinetic studies were conducted for the polymerization of the three PDEGMA-based polymers studied in this work (Supplementary Figures 26-28). For the polymerization of DEGMA to yield PDEGMA, an aliquot was taken after 60 minutes, 120 minutes, 240 minutes, 300 minutes, 360 minutes, and 435 minutes, after which time the polymerization was quenched by submerging the

reaction vial in liquid nitrogen. This yielded the kinetics profile shown in Supplementary Figure 30. For the polymerization of DEGMA from a PEG macro chain transfer agent to yield PEG-b-PDEGMA, an aliquot was taken every 30 minutes for a total reaction time of 3 hours. This yielded the kinetics profile shown in Supplementary Figure 31. For the polymerization of HPMA from a PEG-b-PDEGMA macro chain transfer agent to yield PEG-b-PDEGMA-b-PHPMA, an aliquot was initially taken every hour for three hours. However, due to the slowness of the reaction, the reaction was allowed to proceed overnight so and additional aliquots were taken after 16, 18, 20, and 21.5 hours. This yielded the kinetics profile shown in Supplementary Figure 32.

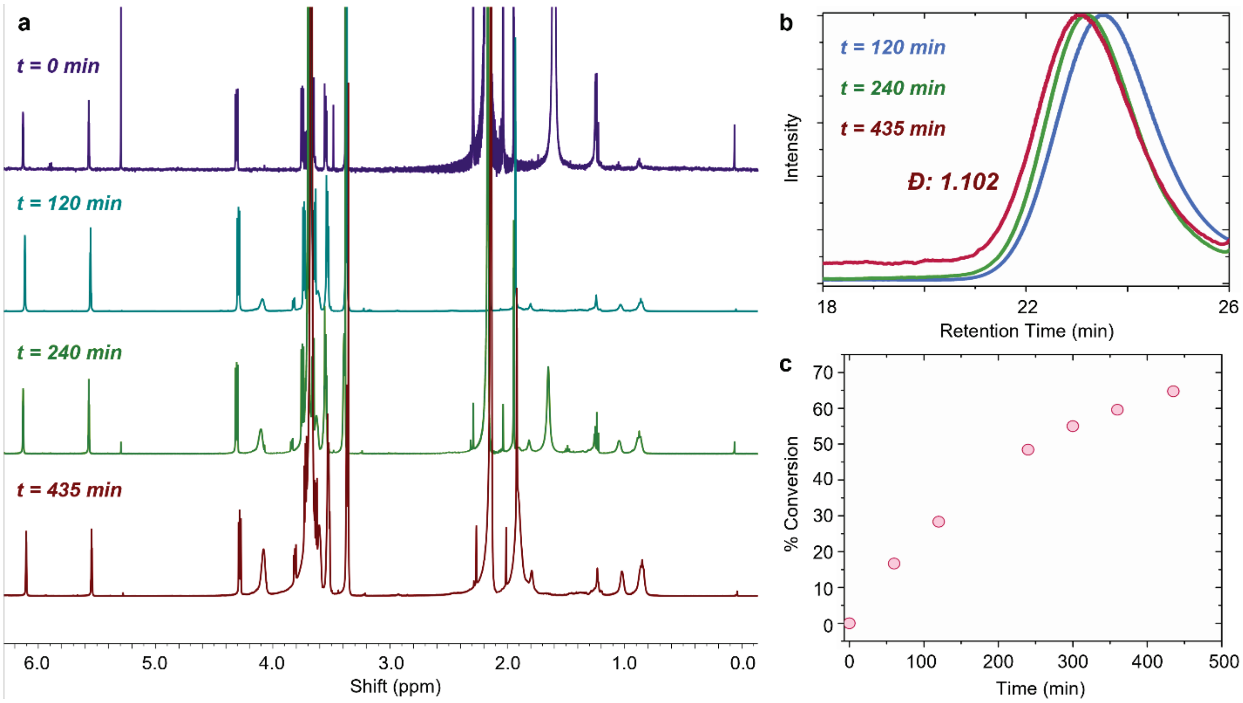

Supplementary Figure 30. Kinetics of the DEGMA polymerization, as depicted in Scheme 1. **a.** ^{1}H NMR spectra of selected aliquots over the course of 435 minute polymerization measured in deuterated chloroform. **b.** SEC-MALS traces of selected aliquots over the course of 435 minute polymerization, showing a polydispersity of 1.102 for the final polymer. **c.** Percent conversion as a function of reaction time, as determined by ^{1}H NMR.

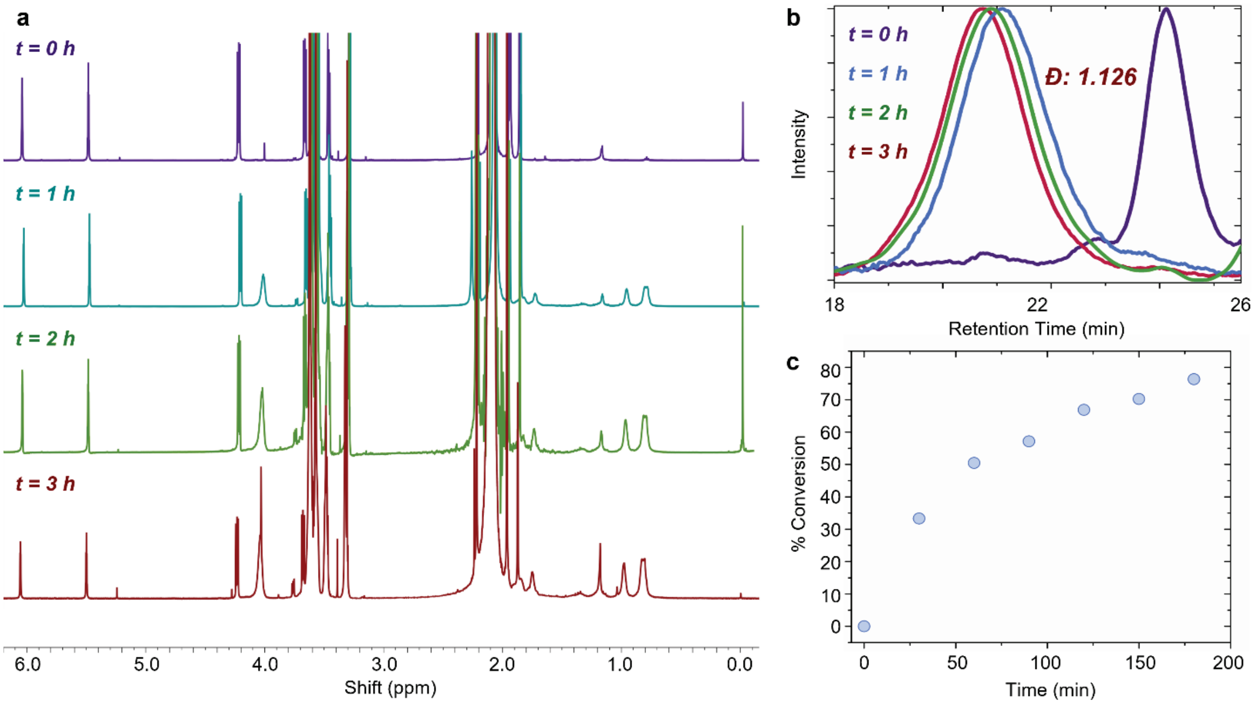

Supplementary Figure 31. Kinetics of the DEGMA polymerization from PEG macro chain transfer agent, as depicted in Scheme 2. **a.** ^{1}H NMR spectra of selected aliquots over the course of 3 hour polymerization measured in deuterated chloroform. **b.** SEC-MALS traces of selected aliquots over the course of 3 hour polymerization, showing a polydispersity of 1.126 for the final polymer. **c.** Percent conversion as a function of reaction time, as determined by ^{1}H NMR.

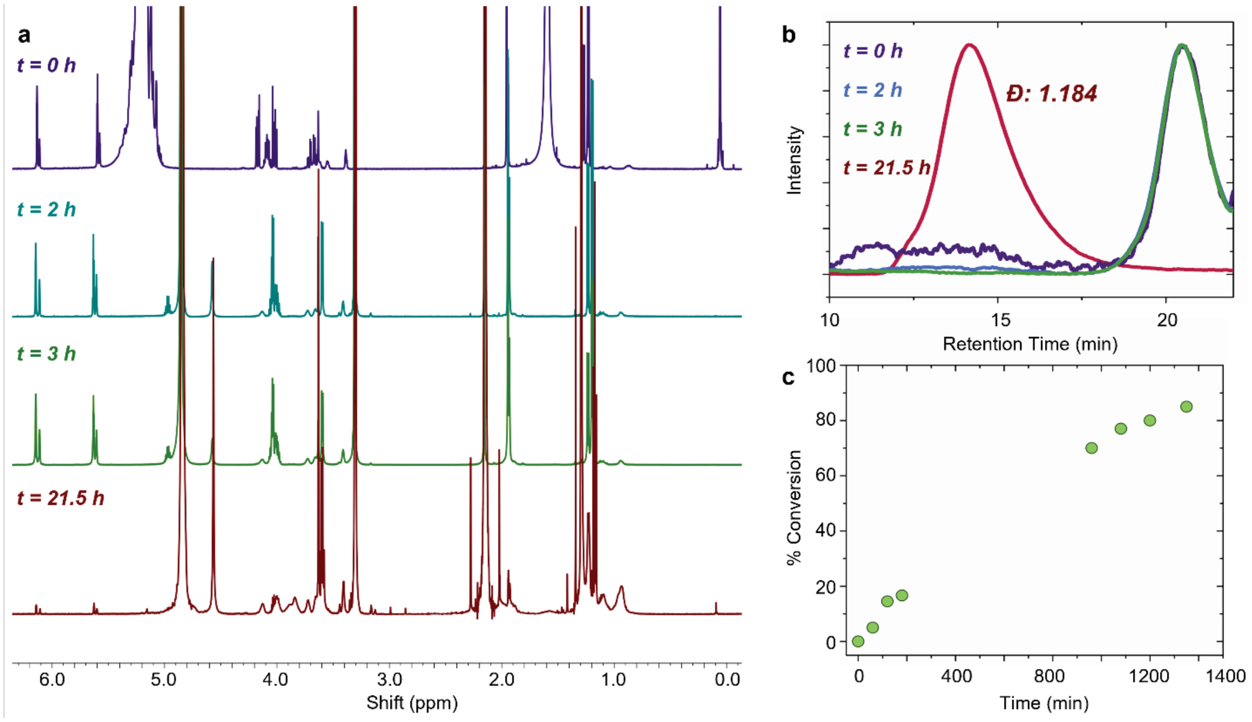

Supplementary Figure 32. Kinetics of the HPMA polymerization from PEG-b-PDEGMA macro chain transfer agent, as depicted in Scheme 3. **a.** ^{1}H NMR spectra of selected aliquots over the course of 21.5 hour polymerization measured in deuterated methanol. **b.** SEC-MALS traces of selected aliquots over the course of 21.5 hour polymerization, showing a polydispersity of 1.184 for the final polymer. **c.** Percent conversion as a function of reaction time, as determined by ^{1}H NMR.

IX. Supplementary References

1. Nishimura T, de Campo L, Iwase H, Akiyoshi K. Determining the Hydration in the Hydrophobic Layer of Permeable Polymer Vesicles by Neutron Scattering. *Macromolecules* 2020, **53**(17)**:** 7546-7551.

2. Keerl M, Pedersen JS, Richtering W. Temperature sensitive copolymer microgels with nanophase separated structure. *Journal of the American Chemical Society* 2009, **131**(8)**:** 3093-3097.

3. Sebastiani F, Yanez Arteta M, Lerche M, Porcar L, Lang C, Bragg RA*, et al.* Apolipoprotein E Binding Drives Structural and Compositional Rearrangement of mRNA-Containing Lipid Nanoparticles. *ACS Nano* 2021, **15**(4)**:** 6709-6722.

4. Valldeperas M, Dabkowska AP, Pálsson GK, Rogers S, Mahmoudi N, Carnerup A*, et al.* Interfacial properties of lipid sponge-like nanoparticles and the role of stabilizer on particle structure and surface interactions. *Soft Matter* 2019, **15**(10)**:** 2178-2189.

5. Parent LR, Bakalis E, Ramírez-Hernández A, Kammeyer JK, Park C, de Pablo J*, et al.* Directly Observing Micelle Fusion and Growth in Solution by Liquid-Cell Transmission Electron Microscopy. *Journal of the American Chemical Society* 2017, **139**(47)**:** 17140-17151.

6. Woehl TJ, Abellan P. Defining the radiation chemistry during liquid cell electron microscopy to enable visualization of nanomaterial growth and degradation dynamics. *Journal of Microscopy* 2017, **265**(2)**:** 135-147.

7. Pietsch C, Mansfeld U, Guerrero-Sanchez C, Hoeppener S, Vollrath A, Wagner M*, et al.* Thermo-induced self-assembly of responsive poly (DMAEMA-b-DEGMA) block copolymers into multi-and unilamellar vesicles. *Macromolecules* 2012, **45**(23)**:** 9292-9302.

8. Konefał R, Spěváček J, Mužíková G, Laga R. Thermoresponsive behavior of poly (DEGMA)-based copolymers. NMR and dynamic light scattering study of aqueous solutions. *European Polymer Journal* 2020, **124:** 109488.

9. Truong NP, Whittaker MR, Anastasaki A, Haddleton DM, Quinn JF, Davis TP. Facile production of nanoaggregates with tuneable morphologies from thermoresponsive P (DEGMA-co-HPMA). *Polymer Chemistry* 2016, **7**(2)**:** 430-440.

10. North SM, Armes SP. Aqueous solution behavior of stimulus-responsive poly (methacrylic acid)-poly (2-hydroxypropyl methacrylate) diblock copolymer nanoparticles. *Polymer Chemistry* 2020, **11**(12)**:** 2147-2156.
