# Supplementary material for: Thermoresponsive polymer assemblies via variable temperature liquid-phase transmission electron microscopy and small angle X-ray scattering
Source: Nat Commun. 2021 Nov 12;12:6568. doi: 10.1038/s41467-021-26773-z (PMC8589985; doi:10.1038/s41467-021-26773-z)
Supplement: Supplementary file 3 — Description of Additional Supplementary Files [file 41467_2021_26773_MOESM3_ESM.pdf]

**Description of Additional Supplementary Files**

File Name: Supplementary Movie 1
Description: LCTEM damage experiment on 5 mg mL−1 PDEGMA imaged at a flux of 2 e− Å−2 s -1 and cumulative fluence of 7.2 x 103 e− Å−2 in pure water. The movie shows one of four irradiated corners, each of which received a fluence of 1.8 x 103 e− Å−2 .

File Name: Supplementary Movie 2
Description: LCTEM damage experiment on 5 mg mL−1 PDEGMA imaged at a flux of 2 e− Å−2 s -1 and cumulative fluence of 7.2 x 103 e− Å−2 in 5% t-butanol in water. The movie shows one of four irradiated corners, each of which received a fluence of 1.8 x 103 e− Å−2 .

File Name: Supplementary Movie 3
Description: LCTEM damage experiment on 5 mg mL−1 PDEGMA imaged at a flux of 2 e− Å−2 s -1 and cumulative fluence of 7.2 x 103 e− Å−2 in deuterium oxide. The movie shows one of four irradiated corners, each of which received a fluence of 1.8 x 103 e− Å−2 .

File Name: Supplementary Movie 4
Description: LCTEM damage experiment on 5 mg mL−1 PDEGMA imaged at a flux of 2 e− Å−2 s -1 and cumulative fluence of 7.2 x 103 e− Å−2 in degassed water. The movie shows one of four irradiated corners, each of which received a fluence of 1.8 x 103 e− Å−2 .

File Name: Supplementary Movie 5
Description: LCTEM damage experiment on 5 mg mL−1 PDEGMA imaged at a flux of 2 e− Å−2 s -1 and cumulative fluence of 7.2 x 103 e− Å−2 in 5% isopropanol in water. The movie shows one of four irradiated corners, each of which received a fluence of 1.8 x 103 e− Å−2 .

File Name: Supplementary Movie 6
Description: LCTEM damage experiment on 5 mg mL−1 PDEGMA imaged at a flux of 2 e− Å−2 s -1 and cumulative fluence of 7.2 x 103 e− Å−2 in 5% dimethyl sulfoxide in water. The movie shows one of four irradiated corners, each of which received a fluence of 1.8 x 103 e − Å−2 .

File Name: Supplementary Movie 7
Description: : LCTEM damage experiment on 5 mg mL−1 PDEGMA imaged at a flux of 2 e− Å−2 s -1 and cumulative fluence of 2.4 x 103 e− Å−2 in pure water. The movie shows one of four irradiated corners, each of which received a fluence of 600 e− Å−2 .

File Name: Supplementary Movie 8
Description: LCTEM damage experiment on 5 mg mL−1 PDEGMA imaged at a flux of 2 e− Å−2 s -1 and cumulative fluence of 2.4 x 103 e− Å−2 in 5% t-butanol in water. The movie shows one of four irradiated corners, each of which received a fluence of 600 e− Å−2 .

File Name: Supplementary Movie 9
Description: LCTEM damage experiment on 5 mg mL−1 PDEGMA imaged at a flux of 2 e− Å−2 s -1 and cumulative fluence of 2.4 x 103 e− Å−2 in deuterium oxide. The movie shows one of four irradiated corners, each of which received a fluence of 600 e− Å−2 .

File Name: Supplementary Movie 10

Description: LCTEM damage experiment on 5 mg mL−1 PDEGMA imaged at a flux of 2 e− Å−2 s -1 and cumulative fluence of 1.2 x 103 e− Å−2 in 5% IPA. Supplementary Movie shows one of two irradiated corners, each of which received a fluence of 600 e− Å−2 .

File Name: Supplementary Movie 11
Description: : LCTEM damage experiment on 5 mg mL−1 PDEGMA in water imaged at a flux of 20 e− Å−2 s -1 showing immediate dehydration of liquid-cell.
